# Fine-Tuning of the Neuropeptide Y1 G Protein-Coupled Receptor by the Tryptophan^6.48^ “Toggle Switch”

**DOI:** 10.1021/jacs.5c07143

**Published:** 2025-12-19

**Authors:** Matthias Voitel, Maik Pankonin, Alexander Vogel, Karl Leitner, Anette Kaiser, Daniel Huster, Peter W. Hildebrand, Albert A. Smith

**Affiliations:** 1 Institute for Medical Physics and Biophysics, 9180 Leipzig University, 04107 Leipzig, Germany; 2 Department of Anesthesiology and Intensive Care, University of Leipzig Medical Center, 04103 Leipzig, Germany

## Abstract

G protein-coupled receptors (GPCRs) transduce extracellular signals into the cell through binding and activation of intracellular effector proteins. Highly conserved residues such as tryptophan W^6.48^ of transmembrane helix 6 can play a role in GPCR activation, where W^6.48^ acts as a microswitch, changing its rotameric state depending on whether the receptor is bound to an agonist or antagonist. However, its exact role is not entirely clear. Here we investigate the role of W^6.48^ in the neuropeptide Y1 receptor (Y1R). Via NMR experiment and molecular dynamics simulations, we find that on the one hand, W^6.48^ exhibits multiple rotameric conformations, where simulations indicate that these are coupled to backbone structure. On the other hand, mutation of W^6.48^ to alanine does not prevent G-protein signaling, and agonist vs antagonist bound Y1R exhibits the same W^6.48^ rotameric state, calling into question whether its core function is to regulate Y1R activation. Further investigation indicates that the W^6.48^ rotameric state restricts microstates accessible by Y1R and impacts backbone dynamics. Using principal component analysis of multiple MD trajectories, we determine the structural similarity between Y1R for the various apo, NPY, NPY/Gi, and antagonist-bound conformational states. We propose a role for W^6.48^ in regulating Y1R binding, in which rotameric changes for W^6.48^ influence ligand binding by favoring backbone structures in apo Y1R that are similar to those observed for the active protein. Mutation of W^6.48^ then does not eliminate these structures but reduces their prevalence. Therefore, W^6.48^ provides “fine-tuning” of Y1R signaling; mutation of the 6.48 position leaves Y1R active but “detuned” such that signaling is still possible, but proper functioning is inhibited by a decrease in ligand binding rate.

## Graphical abstract

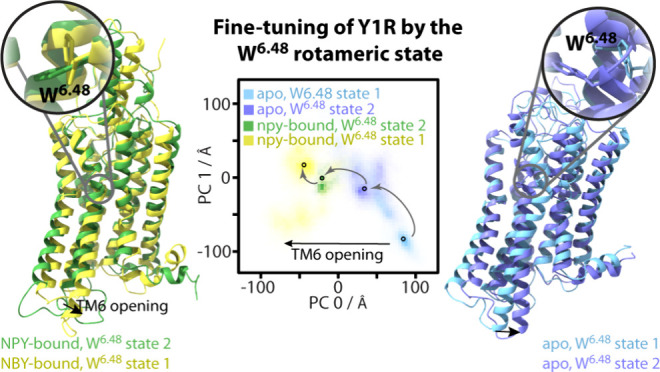

## Introduction

G protein-coupled receptors (GPCRs) are a family of membrane receptors consisting of a seven-transmembrane helix (7TM) structure and play key roles in various important physiological processes.^1^ These proteins are crucial to regulate stimuli between the exterior and the interior of the cell, making them a common target for medicinal drugs aiming to manipulate their signaling.^2^

Naturally, the biochemical and biophysical processes of GPCR signal transmission are intensively researched, revealing insights into conformational and dynamical changes associated with the signal transfer.^3–5^ It is well established that most GPCRs, upon binding of an extracellular ligand, experience an opening of the intracellular G-protein binding site through the rearrangement of the 7TM-helix bundle, mainly an outward tilt of TM6.^6–8^ This enables the binding of the Gα-subunit of heterotrimeric G-proteins and subsequently the exchange of guanosine diphosphate (GDP) to guanosine triphosphate (GTP) triggering downstream signal pathways.^8^ Furthermore, conformational changes of the intra- and extracellular loops are known to be important to regulate ligand binding and signal transfer to G-proteins or arrestins.^9–11^ Overall, it has been shown experimentally as well as by computer simulations that GPCRs in general are very dynamic molecules that undergo a multitude of motions of varying amplitude.^3,12,13^ This is because the receptors exist in a dynamic equilibrium among inactive and active states that may interchange relatively easily due to low energetic barriers between them.^14^ Up to 4 different states have been identified for GPCRs (named S1–S4)^14^ which characterize the inactive state (S1, S2), an active intermediate state where the ligand is bound (S3), and the fully active state where G-protein binding is observed (S4). Different ligands (agonists, antagonists, inverse agonist, etc.) change the populations of the individual states within this ensemble.^14^ Each of the states is also characterized by high conformational flexibility implying that the individual states are not described by a single energy minimum, but rather the energy well of a respective state is characterized by local minima divided by small barriers on the order of *k*_B_ *T*.^15–17^ We refer to the conformations in these local minima as microstates.

This multitude of structural changes is also connected to highly conserved residue motifs within different GPCRs.^12^ Many of these motifs form microswitches, which are residues that are found in significantly different conformations depending on the state of the receptor.^18^ For example, the E­(D)­R^3.50^Y microswitch functions by restricting TM6 outward movement via an ionic lock between TM3 and TM6, thereby keeping the receptor in its inactive state, whereas disruption of the ionic lock by protonation of E^3.49^ enables outward TM6 movement (superscript numbers refer to the Ballesteros–Weinstein nomenclature^19^). Another highly conserved residue is tryptophan 6.48 of the CWxP^7.50^ motif. According to the rotamer toggle switch hypothesis, W^6.48^ and F^6.52^ (called “toggle switch”) change rotamer state during activation.^20^ It was observed that the rotameric states of the aromatic residues in the toggle switch are highly correlated with each other, which is explained by their unusually close proximity due to the highly conserved proline kink (P^6.50^) in TM6. Furthermore, their rotameric conformation was attributed to influence of the proline kink environment indirectly through its neighboring residue at position 6.49. The conformation of the proline kink again was observed to modulate the amount of TM6 movement after breakage of the ionic lock during receptor activation. Consequently, the change of the rotamers of the toggle switch residues was proposed to be an indicator for receptor activation.^20^ Indeed, mutational studies of the β_2_-adrenoreceptor, bovine rhodopspin, muscarinic M_3_ receptor, bradykinin B_2_ receptor, 5-HT_4_ receptor, and ghrelin receptor reinforced the importance of the toggle switch for the activation process of these systems.^20–25^

However, recently published structures of the active muscarinic M_2_ and M_3_ receptors did not show such altered rotameric states of W^6.48^ and F^6.52^ compared to their inactive forms.^26,27^ Similarly, molecular mechanistic mapping of class A GPCRs suggested that W^6.48^ is not a toggle-switch, but rather its position changes due to TM6 rotation and it is also approached by I^3.40^.^28^ This calls the role of W^6.48^ in GPCR activation somewhat into question. Instead, W^6.48^, which is conserved in 68% of class A GPCRs, is rather seen as a reporter for ligand binding. More generally, its importance and functionality for receptor activation seem to differ between individual GPCRs.^29^ Interestingly, new studies found evidence of a potential role of the toggle switch in biased agonism in the adenosine A3 receptor, sphingosine-1-phosphate receptor 1, cannabinoid receptor 1, μ-opioid receptor, and neuropeptide Y2 receptor.^30–34^ Lastly, studies of the μ-opioid and A2A receptor suggest W^6.48^ to be part of an assembly of polar residues, forming a hydrogen bond network with water stabilizing the active conformation. The simulations of A2A receptor also imply W^6.48^ acts as a mostly hydrophobic “lid” that, through transient rotamer exchange, enables water influx and the subsequent formation of the water channel upon activation.^35,36^ In summary, the toggle switch, although not showing a universal mode of function like the ionic lock, is essential to understanding how the individual GPCR systems mediate the signal transfer.

In this study, we use molecular dynamics (MD) simulations combined with NMR spectroscopy to investigate the role of W^6.48^ on the activation of the neuropeptide Y1 receptor (Y1R). We observe variation in the W^6.48^ rotameric state, both experimentally and with simulation, and show a strong correlation of the rotameric state with backbone conformation. Challenging these findings, however, are previous studies that show mutation of W^6.48^ to alanine (W276A) still yields a receptor capable of G-protein signaling.^37^ A careful analysis of the MD simulations suggests a more subtle role for W^6.48^, where different rotameric states shift inactive apo Y1R toward the active conformation, possibly fine-tuning the kinetics of the Y1 GPCR.

## Results and Discussion

### Molecular Dynamics Simulations Reveal Distinct Microstates of Y1R and Transitions Thereof

While the average structure of a GPCR changes depending on its binding state, MD simulations of Y1R furthermore indicate that multiple microstates exist depending on whether it is unbound (apo), bound to NPY, NPY and Gi, or to the UR-MK299 ^37^ antagonist. Such conformational plasticity has also been observed in other GPCRs.^3,38,39^ For Y1R, this is shown in Figure 1, where 12 MD simulations of Y1R (3 each of apo, NPY-, Gi/NPY-, and UR-MK299 bound, totaling 216 μs of simulation time) have been analyzed using a single principal component analysis (PCA) of the protein backbone (C′, Cα, N). PCA sorts motion (more specifically, positional variance) in the protein into independent components, where the lowest-indexed components represent the largest degree of variation. Then, we may examine the extent of structural variation among the trajectories via histograms of the first several principal components. PCA histograms also tend to form clusters so that it is possible to group MD frames into sets of similar structures. Therefore, in Figure 1A, histograms of the two largest principal components (PC0, PC1) are shown, with each row corresponding to a different binding state of Y1R (see SI Figures 1–3, 7–8 for further PCA analysis). Note that because all trajectories were analyzed with the same PCA, positions in the histograms among the various binding states are directly comparable. For each binding state, a different region of the PC0/PC1 histogram is occupied, representing (as expected) differences in the average structure of Y1R depending on its binding state. However, within each binding state, there are also several microstates, i.e., structural variation occurring within the binding states, in this case typically near the submicrosecond/microsecond time scale. For each binding state, the structures are grouped into 4–6 clusters, where the middle of each cluster is marked in Figure 1A (black triangles), along with the percent of the total time spent in each cluster. The frame from the trajectory closest to the middle of each cluster is shown in Figure 1B; protein regions with more structural variation within the binding state are highlighted in red.

**Figure 1:**
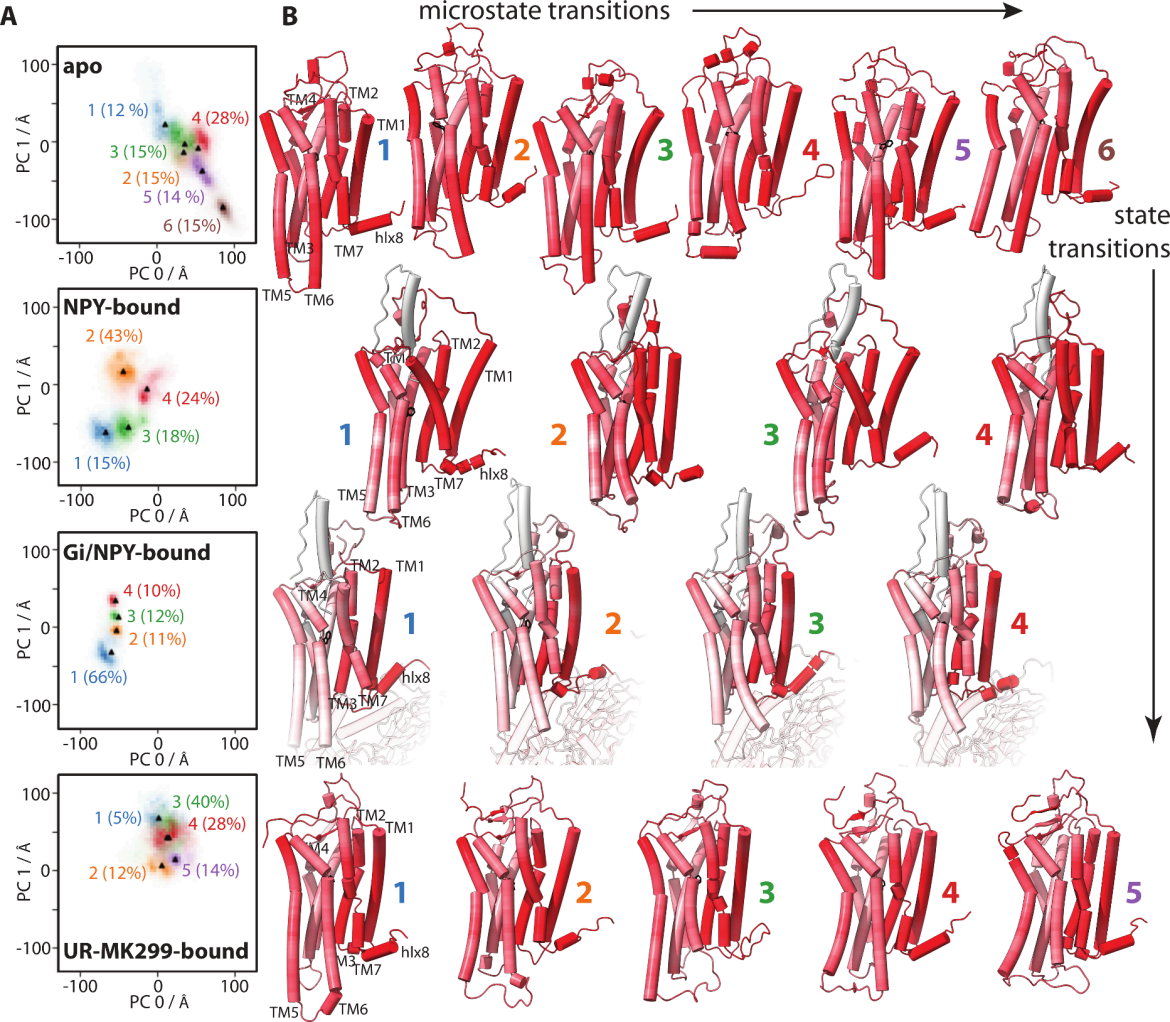
Comparison of structures as a function of the binding state and microstate. Panel A shows histograms of the first two principal components for each binding state of Y1R, where the histogram has been grouped into 4–6 clusters. Heavily overlapped clusters are better resolved in higher dimensions of the PCA (see SI Figure 2 and SI Figure 3). Points marked in the histograms correspond to the middle of each cluster, for which the closest matching frame is extracted from the MD trajectory and displayed in panel B. Structures are colored from white to red, where red regions indicate a higher root-mean-square fluctuation of the backbone within the binding state (coloring intensity corresponds to the logarithm of the RMS). 3D protein representations were created with ChimeraX.^40^

The structural variation among microstates for each binding state is extensive, where the differences within a given binding state for PC0/PC1 are sometimes larger than the differences between binding states. We wonder, then, whether we can capture evidence of these microstate transitions experimentally and determine how modulation among microstates is regulated. However, characterizing the structural variation for each binding state of Y1R poses particular challenges. First, it has not been possible to determine the Y1R apo structure by classical structural biology methods, so no structure is available. Structures obtained via X-ray crystallography or cryo-EM do not represent the full dynamics of the bound receptor in its native environment. Therefore, solid-state NMR is an ideal experimental method, where dynamic proteins may be studied in fluid membranes without requiring crystallization, and furthermore, the chemical shift in NMR is a strong reporter of protein structural changes. For technical reasons, NMR acquisition requires the freezing of the sample. This can freeze states that are normally in exchange, which has the advantage that we may then observe those states as separate resonances. However, selective isotope labeling is usually required for such large and dynamic membrane proteins in solid-state NMR, especially if significant structural variation is present, in order to simplify the resulting spectra. Cell-free expression of proteins allows residue-specific isotope labeling for NMR,^15,41,42^ where tryptophan residues would be an ideal choice for isotopic labeling, given only five residues are found in Y1R, thus improving NMR resolution and simplifying assignment. Furthermore, the intricate role of W^6.48^ in Y1R activation makes it an interesting target for investigation. As a first step, we would like to verify that W^6.48^ rotameric dynamics indeed couple to backbone dynamics in our MD trajectories.

We begin by determining residues for which the NMR chemical shift might indicate backbone configurational changes. Backbone structural variation may be coupled to GPCR side-chain dynamics, typically seen for GPCR microswitches.^28,43^ To search for side chains coupled to backbone motion, we analyze the three apo Y1R trajectories, for which we expect the greatest structural variation, and correlate side-chain rotameric states with the PCA clusters shown in Figure 1A (top). We perform an entropy-based cross correlation analysis,^44^ which assigns discrete states to the backbone (via the cluster index in Figure 1) and to the side chains (via rotameric state, where valine has 3 possible rotameric states, leucine has 9, tryptophan has 6, etc.). Correlation is determined by comparing the entropy of the backbone and side chain separately ( $S=-k_{b}\underset{i}{\sum }p_{i}\log\left(p_{i}\right)$ , where *i* runs over the clusters or possible rotameric states, and the *p*_*i*_ are the populations, which sum to 1), to their total combined entropy. Then, if *S*_BB_ + *S*_SC_ = *S*_total_, the correlation is 0, but if *S*_BB_ = *S*_SC_ = *S*_total_, the correlation is 1 so that we may define an entropy-based correlation coefficient, ECC = 2­(*S*_BB_ + *S*_SC_ – *S*_total_)/(*S*_BB_ + *S*_SC_).

The results of this correlation are shown in Figure 2A, with the extent of correlation mapped onto the molecule in Figure 2B. We see that a number of residues in the receptor correlate strongly with backbone clusters in apo Y1R. At the extracellular side, N181^ECL2^ and Y176^4.63^ exhibit high correlation. In the GPCR core, side-chain rotameric states of W276^6.48^ (Figure 2C) are indeed among the most correlated in this analysis. Furthermore, residues F272^6.44^, L131^3.43^, and Y231^5.58^ extend from W^6.48^ toward the intracellular side of Y1R, where Y231^5.58^ is another important microswitch that coordinates arginine of the E­(D)­R^3.50^Y motif, highlighting a possible connection between the lipid binding pocket and the G-protein binding site. Then, given W^6.48^’s importance as a microswitch in GPCRs, and the limited number of tryptophans in Y1R (5 residues), we proceed with tryptophan as a probe for ^13^C NMR investigations.

**Figure 2:**
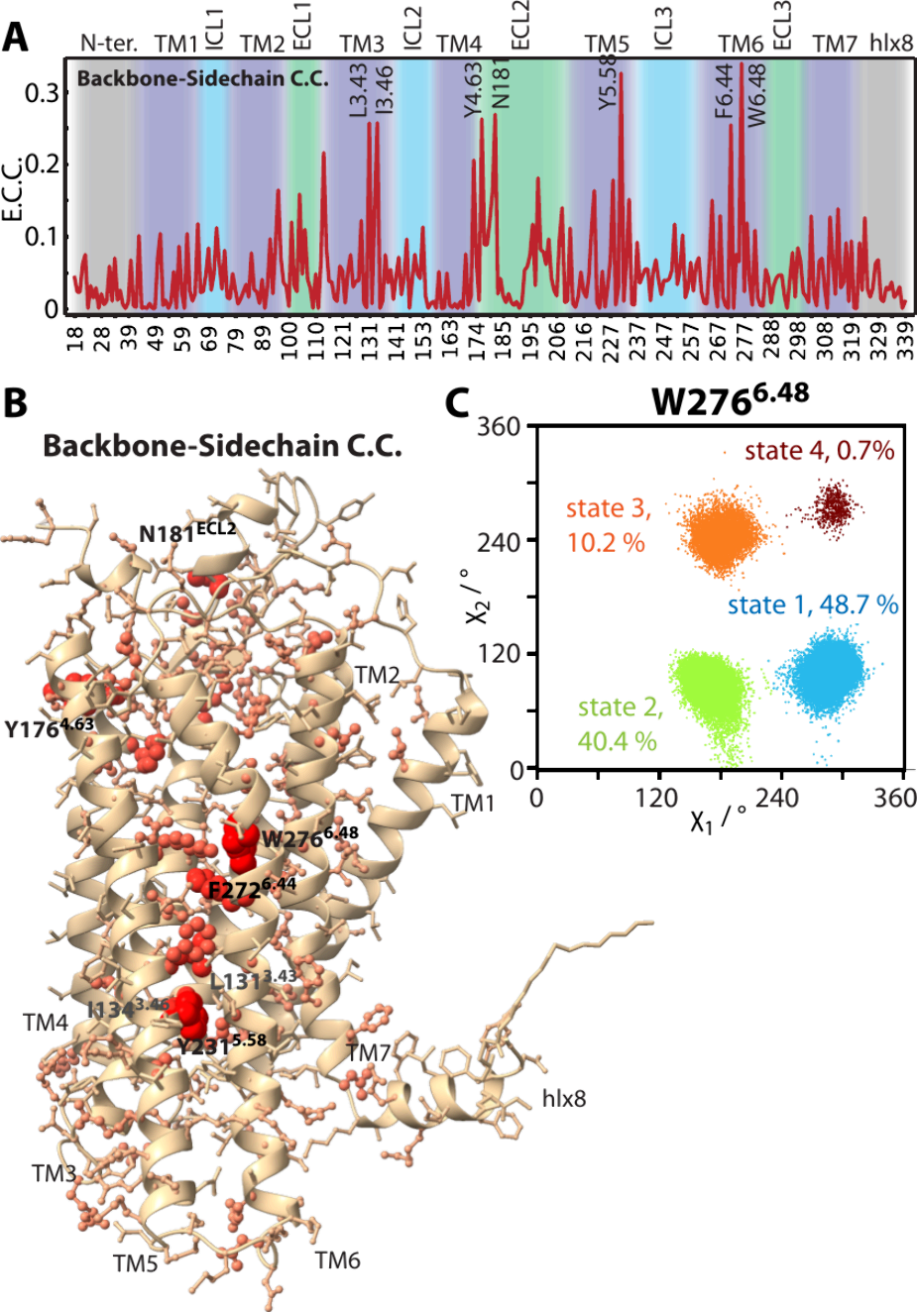
Correlated backbone and side-chain motion in apo-Y1R. Part A shows the entropy-based cross-correlation between the backbone clusters (Figure 1A) and side-chain rotameric states (alanine, proline, and glycine are excluded, since they do not have discrete rotameric states). Part B plots the cross-correlation onto the Y1R structure, where side chains indicated with larger spheres are more correlated. Part C plots the χ_1_ vs χ_2_ side chain rotameric angles in order to show the four rotameric states for W^6.48^.

### Experimental Verification of Multiple Conformations: The W^6.48^ Microswitch

To this end, we prepared samples of Y1R via cell-free expression with ^13^C isotope labeling only on the tryptophans. Cell free synthesis, refolding, and reconstitution were carried out as described previously.^15,41,42^ The receptor construct was modified by an additional H-Tag (KPYDGP) between the initial methionine and Asn2 to enhance the expression yield^45^ and a C-terminal Histidine-Tag (PGGGSH_6_) for purification (full sequence given in SI Figure 4). The *E. coli*-based cell free expression yielded between 0.2 and 1.4 mg of unfolded Y1R per 1 mL of reaction mixture within 24 h depending on conditions and the respective mutant. After expression, the precipitated protein was solubilized with 50 mM DTT and 15 mM SDS and purified using affinity chromatography via the histidine-Tag. In order to obtain a functional receptor, an established protocol for receptor reconstitution in DMPC membranes was performed with 60–80% folding yield.^46^

The samples of Y1R reconstituted into DMPC membranes were investigated by ^13^C MAS NMR. Figure 3B shows the Cα–Cβ regions of the ^13^C–^13^C DARR^47^ NMR spectra with a short mixing time of 10 ms, which should yield signals resulting from the five tryptophans found in wild-type Y1R. A broad spectrum is obtained, and while there are clearly multiple signals, separating and assigning them to individual tryptophans is not possible. Because of this, Y1R mutants were used to reduce the spectral complexity and assign the resonances. We prepared a number of Trp mutants (Trp replaced by Phe) including double (Δ2-Trp), triple (Δ3-Trp), and quadruple (Δ4-Trp) mutants. To verify NPY binding, we performed ligand binding assays showing similar EC_50_ values for the Δ3- and Δ4-Trp mutants with the wild-type receptor (Figure 4A). This confirms that *in vitro*, the mutations do not impair NPY binding ability. Nevertheless, the Δ4 mutant represents a very drastic modification of the receptor, and we resorted to cell assays and fluorescent microscopy in HEK293 cells to investigate the impact on protein function. IP1 accumulation assays (Figure 4B,C) confirmed that the Δ2- and Δ3-Trp mutants still showed reasonable activity with minimal change to the EC_50_ values (Supporting Information Table 3), but for Δ4-Trp, virtually all activity is lost. Fluorescence microscopy of eYFP-tagged receptors (SI Figure 5) indicates that this loss of activity may result from failure of the Δ4 mutant to be incorporated into the cell membrane rather than significant structural changes. Nonetheless, we will keep in mind that signaling activity is not established for the Δ4 mutant.

**Figure 3:**
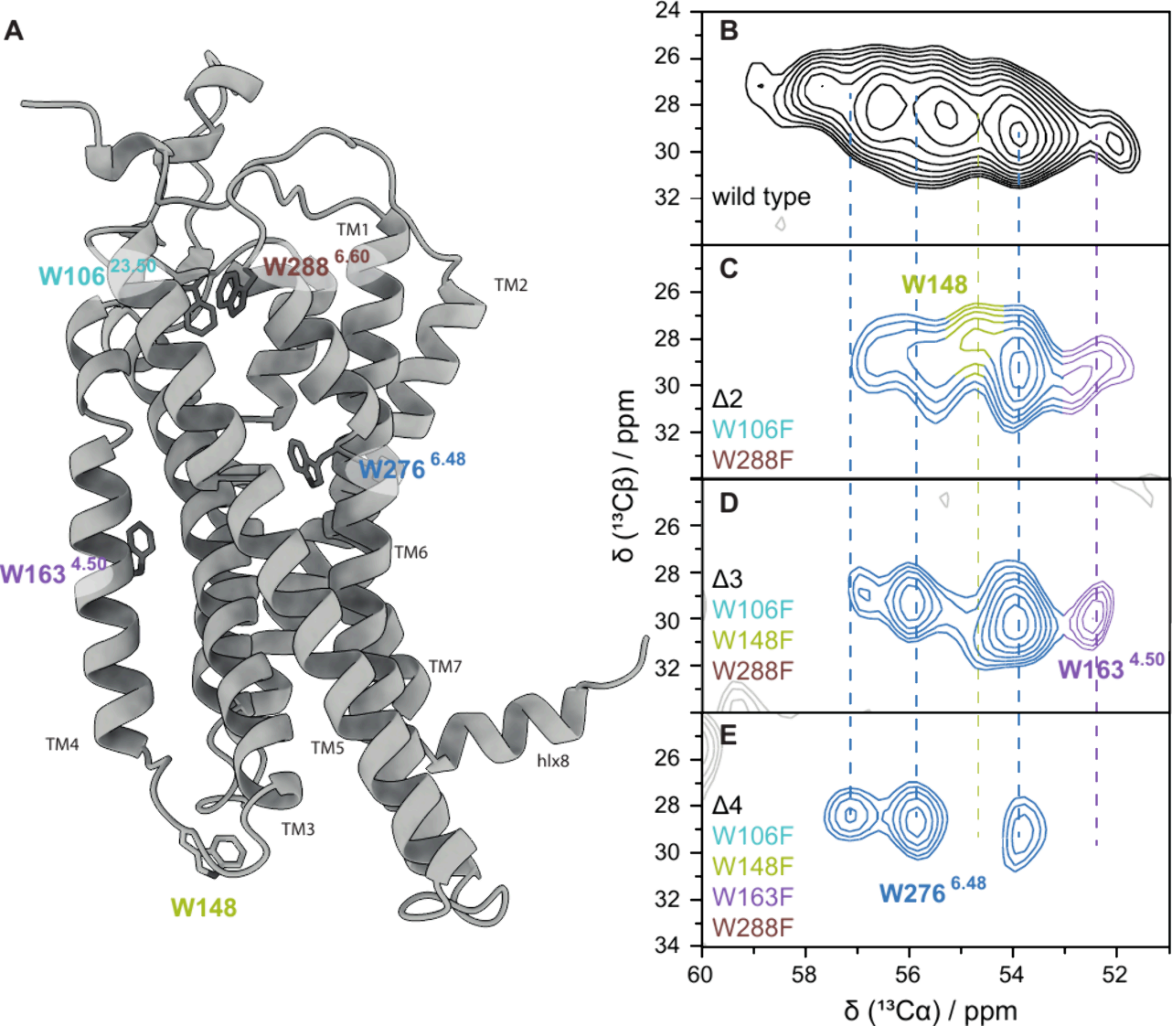
Solid-state NMR of the U-^13^C/^15^N-Trp Y1R WT and mutants. Part A shows the backbone structure of Y1R with tryptophan positions indicated in turquoise (W106), yellow (W148), purple (W163), blue (W276), and brown (W288). Part B shows the Cα–Cβ region of a ^13^C–^13^C DARR spectrum for wild-type Y1R. Part C shows the double mutant, Δ2-Trp (W106F, W288F). Part D shows the triple mutant, Δ3-Trp (W106F, W288F, W148F), and Part E shows the quadruple mutant, Δ4-Trp, for which only W276^6.48^ remains.

**Figure 4:**
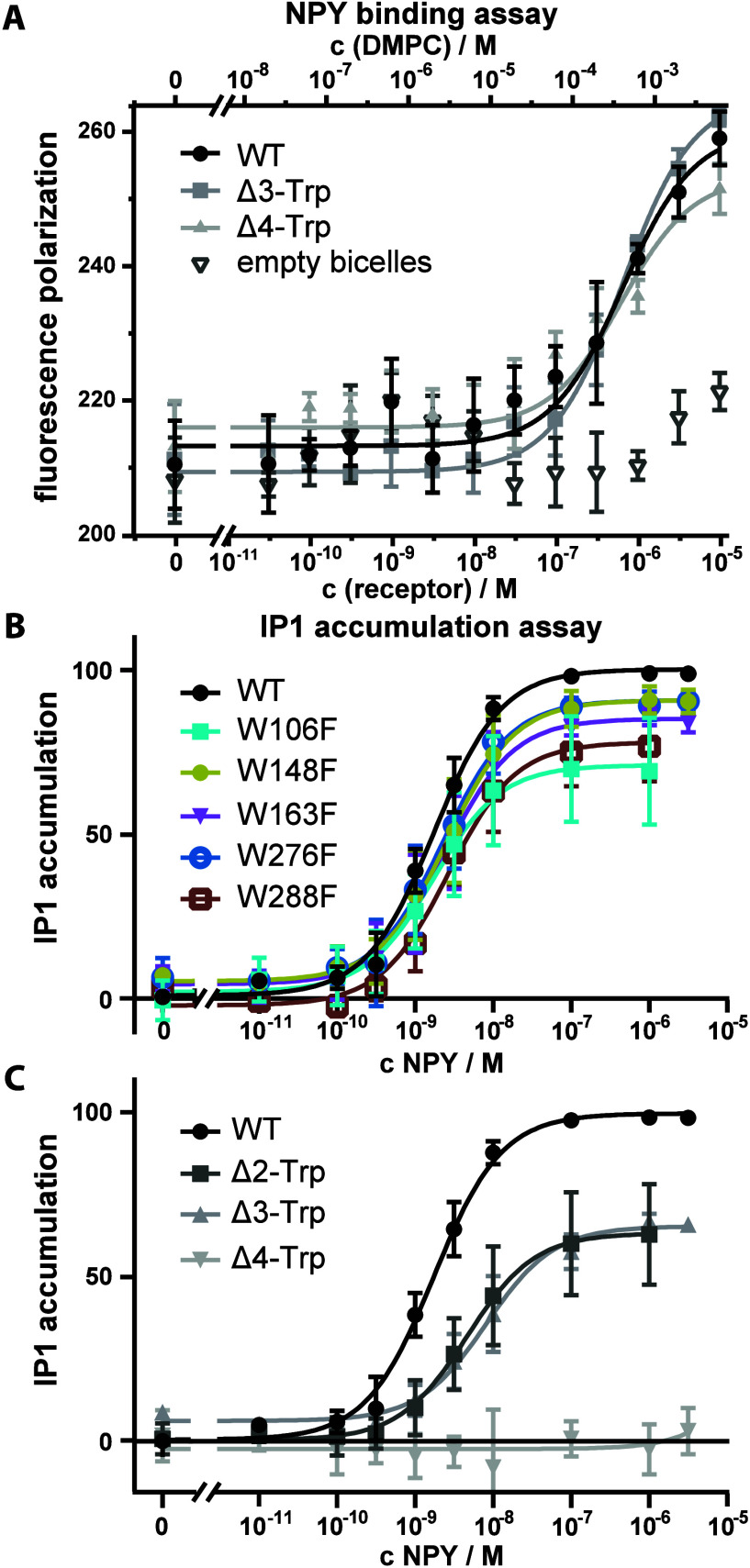
NPY-binding and functionality assays of tryptophan deficient Y_1_R mutants in HEK293 cells. Part A shows binding of [Dpr^22^(Atto520)]-pNPY to the constructs of the Y1R wild-type (black dots), Δ3-Trp (medium gray squares), and Δ4-Trp mutant (light gray, closed triangles) used for NMR samples. Binding is indicated by an increase in fluorescence polarization. Empty bicelles (dark gray, open triangles) are DMPC/DHPC bicelle preparations without receptor. EC_50_ values were determined from the fitted function for Y1R wt (650 ± 140 nM), Δ3-Trp (660 ± 50 nM), and Δ4-Trp (630 ± 330 nM). Parts B and C show IP1 accumulation reporting on G-protein downstream signaling. Part B shows the effect of individual mutations to phenylalanine on each of the five tryptophans (W106F cyan, W148F olive, W163F magenta, W276F blue, and W288F brown) compared to the WT receptor (black). Part C shows the multiple mutants Δ2 (W106F, W288F, dark gray), Δ3 (W106F, W148F, W288F, medium gray), and Δ4 (W106F, W148F, W163F, W288F, light gray). Data are the mean and one standard deviation of at least two independent biological experiments each conducted in triplicate.

We next conducted ^13^C–^13^C DARR NMR experiments for signal assignment, shown in Figure 3C–E. While these mutant proteins allowed assignment of W148^ICL2^ and W163^4.50^, for W276^6.48^ an interesting situation was found: The quadruple mutant, which should leave only W^6.48^ remaining to yield one signal, shows three clearly separate peaks, apparently indicating three distinct conformations of W^6.48^ in apo-Y1R. However, if Δ4 Y1R is bound to its ligand, neuropeptide-Y (NPY), only one peak is observed (Figure 5A). Because Δ4 Y1R did not exhibit signaling activity, we verified these results with Δ3 Y1R, where four peaks were observed: three of those chemical shifts matching the Δ4 spectrum, and the remaining peak corresponds to W163. Similarly, NPY-bound Δ3 Y1R shows two peaks, with one arising from W163, thus confirming the three distinct W276^6.48^ conformations on active protein for apo Y1R, and one conformation for NPY-bound Y1R.

**Figure 5:**
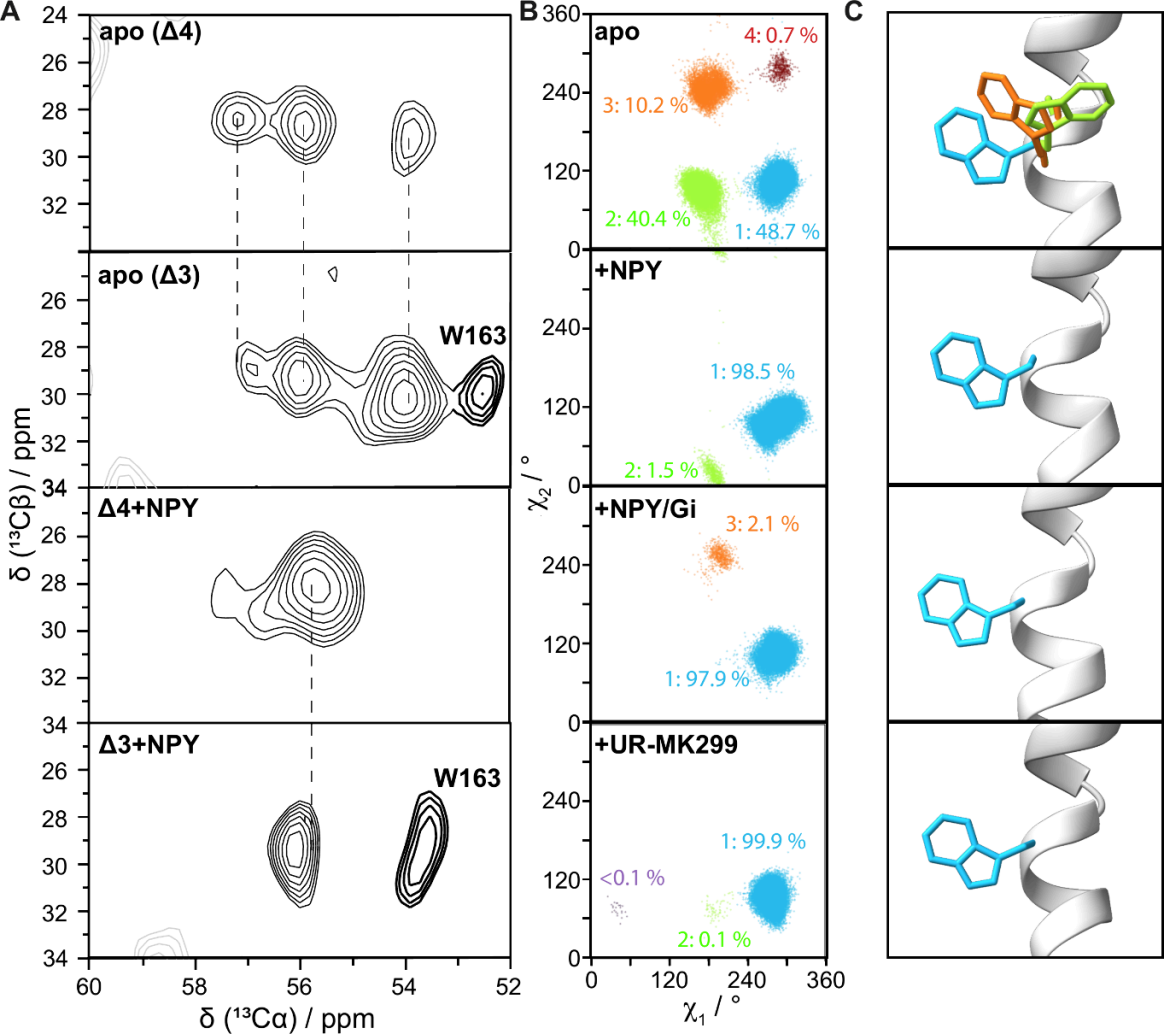
Comparison of the W^6.46^ dynamics of different Y1R binding states observed by MD and NMR. Part A shows ^13^C–^13^C DARR spectra of Δ4-Trp Y1R, in its apo form and bound to NPY. Since Δ4-Trp Y1R may be inactive, we also compare spectra of Δ3-Τrp Y1R in its apo and NPY bound form. Dashed lines are a guide to compare peak positions and are aligned with the center of the resonances in the apo (Δ4) spectrum. Scatter plots in part B show rotameric angles observed in MD simulations for each apo, NPY-bound, NPY/Gi-bound, and UR-MK299 bound Y1R. Colored scatter points cluster together the major rotameric populations, which correspond to the 3D representations shown in part C. Populations below 5% are not expected to yield sufficient signal to be observable with NMR.

To better understand the origin of the signals observed for Y1R by NMR analysis, we consider the MD simulations of apo, NPY-, NPY/Gi-, and UR-MK299-bound Y1R. In Figure 5B, we plot rotameric (χ_1_, χ_2_) angles for W^6.48^, where we indeed observe three rotameric states for apo Y1R (a fourth state accounts for only ∼1% of time points in the trajectory), consistent with the three experimental signals. In agreement with the experiment, NPY-bound Y1R only exhibits one significant rotameric population (∼98%). NPY/Gi- and antagonist-bound Y1R also show only one strong rotameric population.

Interestingly, while our results indicate that the W^6.48^ rotameric state is sensitive to ligand binding and furthermore that the NMR chemical shift is a good reporter on the rotameric state, we see the same W^6.48^ rotameric state in active (NPY/Gi-bound) and inactive (UR-MK299-bound) Y1R, calling into question as to whether it plays a role in activation, or simply reports on fluctuations between states and microstates in Y1R. Previous studies also indicate an unclear role, where W^6.48^276A mutants are still able to transduce G-protein signals.^37^

This leaves us with a conundrum as to the relevance of W^6.48^ to the Y1R function. On the one hand, W^6.48^ is a conserved position, shown to play an important role as a “toggle switch” in other GPCRs. Furthermore, its rotameric state is correlated to backbone conformation in apo Y1R, and indeed, we see experimentally that distinct rotameric states of W^6.48^ do occur. On the other hand, it appears that active and inactive Y1R have the same rotameric conformation of W^6.48^, and replacing it with phenylalanine (Figure 4A,B) or alanine^37^ neither prevents NPY binding nor Y1R G-protein signaling.

### Structural Analysis of W^6.48^ Rotameric States

We analyze our MD simulations to understand in greater detail how the rotameric state of W^6.48^ affects the overall dynamics of the Y1 receptor. Considering again only apo Y1R simulations, we separate the PCA by the rotameric state of W^6.48^, resulting in the four histograms shown in Figure 6A (these histograms then sum to the histogram in Figure 1A, top). Different positions in the PCA histograms represent different Y1R backbone structures, so that we will be able to use the PCA as a function of W^6.48^ rotameric state to determine how the backbone structure depends on that state. We start by noting that state 1 accounts for about half of all MD frames, and samples a wide range of conformations whereas states 2–4 are increasing less populated and more restricted in sampling space. Figure 6B shows the W^6.48^ conformation and nearby (4 Å) residues for selected frames (positions of those frames in the histogram are marked in part A).

**Figure 6:**
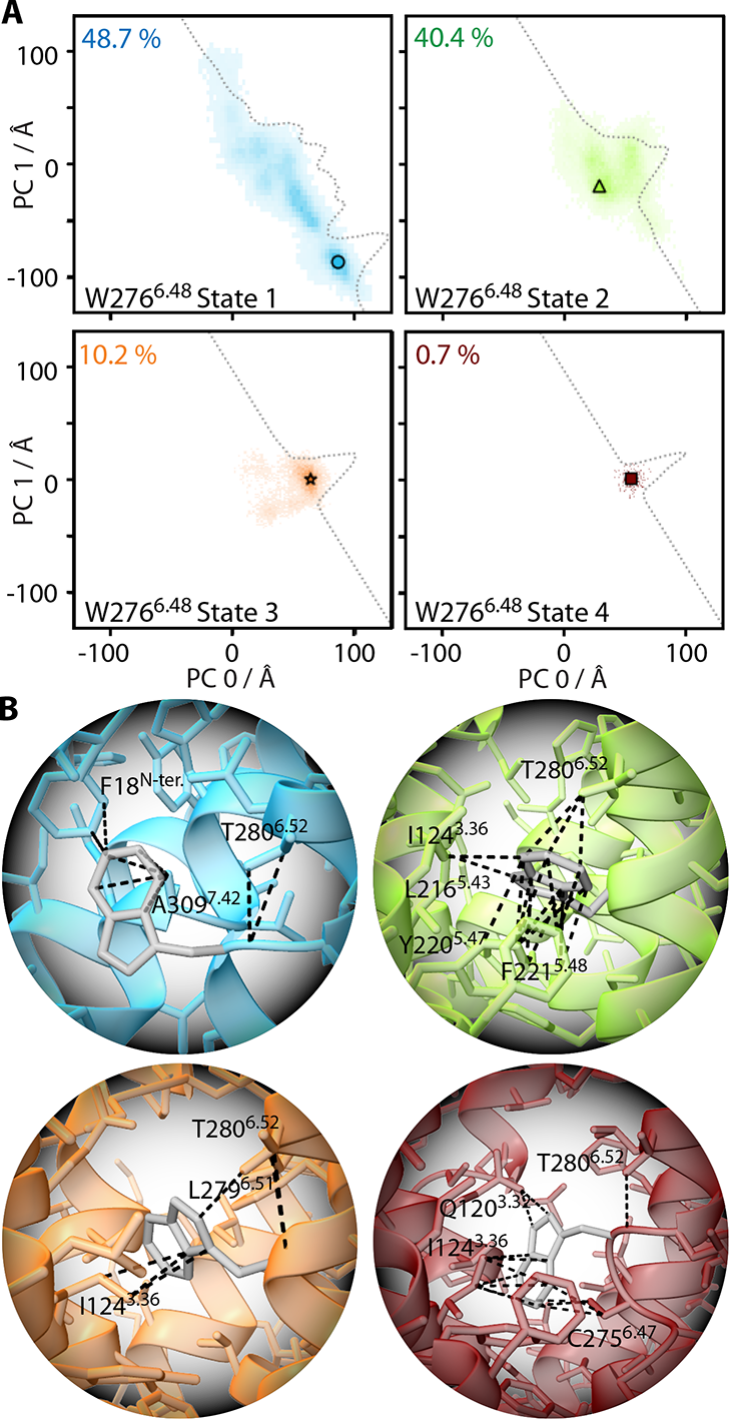
Structural analysis of Y1R as a function of the W^6.48^ rotameric state. (A) Histograms of apo Y1R, showing only PCs for a particular rotameric state of W^6.48^ (same PCA as Figure 1). The percentage in each plot indicates the fraction of all trajectories spent in each rotameric state. Traces are one-dimensional histograms, calculated at a 60° angle to PC 0 and PC 1, to better visualize the shift in population distribution for each rotameric state. Part B shows the orientation of the W^6.48^ side chain for each state for a selected frame (markers in part A), as well as side chain contacts within 4 Å.

Then, we see that each W^6.48^ rotameric state favors different backbone conformations. In particular, when in state 1, larger values of PC 0 and smaller values of PC 1 represent the highest population of backbone conformations, whereas states 2–4 shift PC 0 toward smaller values and PC 1 toward larger values. From SI Figure 7, we see that larger values of PC0 indicate an inward tilting of TM6, whereas smaller values of PC1 indicate a leftwards shift of TM5 and TM6 (when viewed from the side with TM5-TM7). Then, for example, W^6.48^ state 1 exhibits the greatest closure of TM6 for apo Y1R.

Since the W^6.48^ rotameric state remains fixed for extended periods, we also may compare dynamics for each rotameric state (excluding state 4, which is not populated for long enough to evaluate the dynamics). We take 6.7 μs chunks of the trajectories (marked in Figure 7A), where only one rotameric state is sampled (with infrequent, short traverses to other rotameric states), and analyze these with detector analysis,^48^ where we encode motion in the ∼2 μs time scale window in Figure 7B onto the structure for residues in helical regions (loop residues are excluded, where large amplitudes prevent visualization of the helical motion). We also perform correlation analysis^49^ of W^6.48^ to surrounding residues (Figure 7C). Clear dynamics differences are seen among the three rotameric states. First, we see that rotameric state 1 is the only state that exhibits significant motion on the intracellular side of transmembrane helices TM5 and TM6, where the G-protein binds. This movement could be important to allow the opening of the intracellular side of Y1R for G-protein binding. Interestingly, state 3 allows more motion on the extracellular ligand binding side of Y1R than states 1 and 2. We speculate that such dynamics could assist in promoting the NPY binding. We also note that the motion in state 3 is highly correlated with W^6.48^, which is not seen in the other two states (although weak correlation exists in state 1).

**Figure 7:**
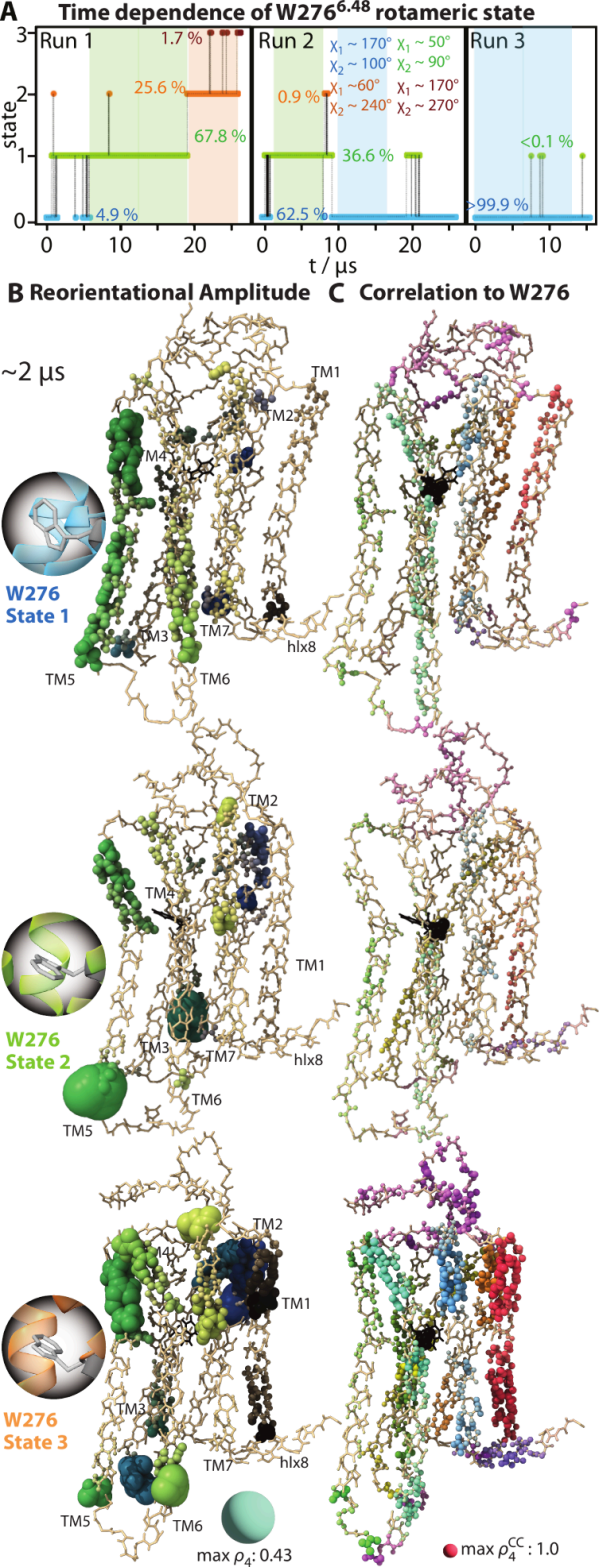
Backbone dynamics of Y1R as a function of the W^6.48^ rotameric state. Panel A shows the rotameric state of W^6.48^ as a function of time in each of the three apo trajectories, with the percent of time in each state and the average χ_1_ and χ_2_ angles for each state indicated on the plots. Structural plots in parts B and C show dynamics as a function of rotameric state, where 6.7 μs chunks of the trajectories were analyzed, within which the rotameric state is mostly fixed. These chunks are indicated as highlighted regions in part A. Color coding is the following: state 1, blue; state 2, green; state 3, orange. Part B plots the reorientational amplitude of motion (from 0 to 1) for residues in helices (intra- and extracellular loops are excluded due to large dynamics that mask the helix motion). Part C plots the correlation of reorientational motion to the motion of W^6.48^ (from 0 to 1, with 1 being fully correlated). Motion is within the ∼2 μs time scale window.

Each rotameric state of W^6.48^ has different impacts on the structure and dynamics of the Y1R. We use the trajectories of NPY- and NPY/Gi-bound Y1R as ideal reference structures for active states, where NPY/Gi-bound Y1R should be fully active (state S4), and NPY-bound Y1R (state S3) should be on-path toward the active structure, and antagonist-bound Y1R should be inactive. In Figure 1, we see that PC 0 is a good indicator for Y1R activation, where NPY/Gi-bound Y1R exhibits the smallest values for PC 0, and NPY-bound has second smallest, with more variation toward larger values. Indeed, PC 0 primarily indicates the outward tilt of TM6 from the center of the receptor (SI Figure 7). Then, apo clusters 1–3 represent the closest approach to an active structure by apo Y1R, whereas cluster 6 is the most inactivated, due to TM6 closure and also a shift of TM6 sideways toward TM5 (the RMSD between the center of all 19 clusters is calculated in SI Figure 8).

Y1R shows measurable but low basal activity in regard to Gi signaling^50^ and arrestin-2 recruitment^51^ and so should approach but not access bound conformations (state S3). NPY-bound cluster 4 represents the closest approach to apo Y1R, whereas NPY-bound clusters 1 and 2 are closer to the NPY/Gi-bound structures (Figure 1A). Interestingly, while Y1R’s most inactive conformation, corresponding to apo cluster 6, is structurally very different than NPY-bound Y1R, antagonist-bound Y1R is structurally similar to apo clusters 1–4, with W^6.48^ in rotameric state 1 (Figure 1A, Figure 6A). This similarity may be because apo trajectories were initialized in apo cluster 1 (biasing simulations toward those structures), but regardless, it shows that apo Y1R accesses structures with greater intracellular closure of TM6 than antagonist-bound Y1R.

A comparison of active and inactive structures gives an important hint of the role of W^6.48^ in Y1R signaling. We note that in Figure 6A, conformations were affected by the rotameric state of W^6.48^, where W^6.48^ rotameric states 2 and 3 shift the backbone populations toward structures most similar to NPY backbone PCA cluster 4 (Figure 1A, second row). The presence of W^6.48^ rotameric states 2 and 3 then moves the Y1R thermal equilibrium toward conformations more structurally similar to NPY-bound Y1R, potentially accelerating NPY binding to Y1R. However, if this is the case, then presumably Y1R should be able to bind NPY while W^6.48^ is in state 2 or 3, and the corresponding backbone structure should be similar to that of apo Y1R. We already saw that NPY-bound Y1R accesses W^6.48^ rotameric state 2 in Figure 5B (second row), but we have not investigated the corresponding backbone conformations via PCA. Therefore, Figure 8B plots a histogram of apo Y1R with W^6.48^ in rotameric state 2, overlaid with NPY-bound Y1R, also in rotameric state 2. Remarkably, the region of the histogram occupied by NPY-bound Y1R with W^6.48^ in state 2 corresponds to its closest approach to conformations accessible by apo Y1R, and these apo conformations also occur when W^6.48^ is in rotameric state 2.

**Figure 8:**
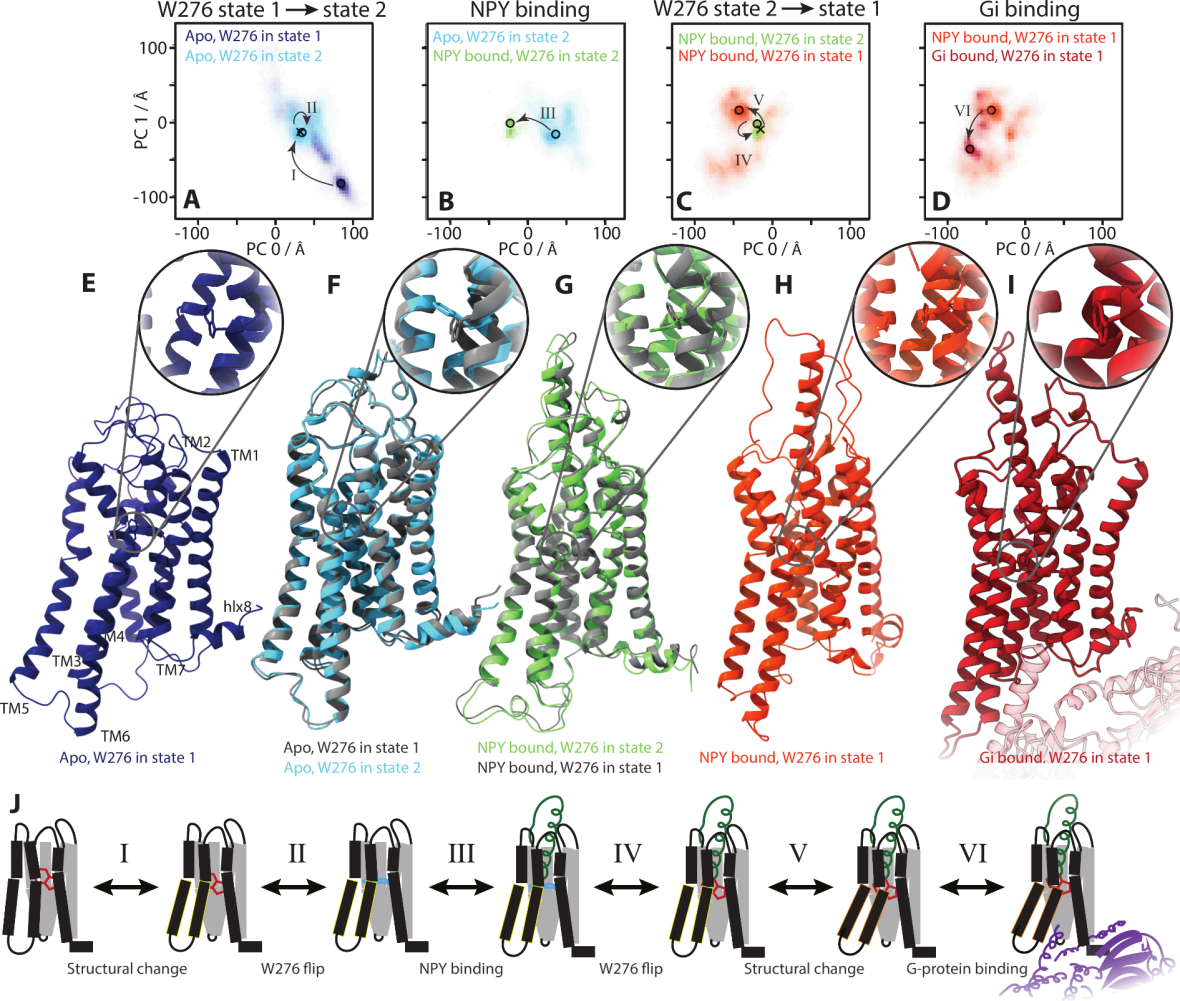
Proposed activation process of Y1R. Each plot overlays two PCA histograms, with each histogram corresponding to a particular trajectory and rotameric state of W^6.48^ (same PCA as Figure 1 and Figure 6). A possible pathway from apo Y1R to Gi-bound Y1R is outlined at the top, including rotameric flips of W^6.48^, where scatter points correspond to the structures shown at the bottom. The scatter points and proposed transitions are as follows: in part A, a structure far from activation in W^6.48^ state 1 (dark blue circle, structure in part E) transitions (I) to a structure closer to activation (cross, gray in part F), followed by flipping (II) of W^6.48^ to state 2 (light blue circle, light blue in part F). This structure is marked again in part B, where it transitions to (III) upon NPY binding to the green circle (green in part G), and W^6.48^ remains in state 2. This structure is marked again in part C, where W^6.48^ flips (IV) back to state 1 (cross, gray in part G) followed by transitioning (V) to a more frequently observed NPY-bound structure (orange circle, shown in part H). This structure is shown again in part D, where additional structural changes occur upon Gi binding (VI), resulting in the structure indicated by a red circle (structure in part I). The complete set of transitions is illustrated as a scheme in part J, where roman numerals correspond to those in parts A–D. Trp rotameric changes are highlighted by changing the color of the Trp.

### A Model of Successive Conformational Changes That Lead to Y1R Activation

This observation leads us to propose a step-by-step process for activation of the Y1R, from inactive Y1R to NPY/Gi-bound and fully active Y1R, noting the role that the W^6.48^ rotameric state plays in that activation. We illustrate this process in Figure 8, where we begin with the apo conformation and W^6.48^ in state 1, having a large PC 0 value (Figure 8A), corresponding to the TM6 closure (Figure 8E). TM6 can then undergo partial opening on the intracellular side (decreasing PC 0) and also a rightward shift (PC 1), marked as step I (see SI Figure 10 for an overlay of the first two structures). This conformation is shown in gray in F, where a subsequent flip of the W^6.48^ rotamer to state 2 (step II) stabilizes this partial opening of TM6, and prevents return of the apo Y1R to the initial conformation in E. The corresponding structure is shown in F (cyan) and occurs one frame (1 ns) after the gray structure, where the W^6.48^ flip does not significantly modify the backbone structure. NPY may then bind, bringing about step III in step B, corresponding primarily to an additional opening of TM6. Backbone structures for NPY-bound Y1R with W^6.48^ in state 2 are highly restricted (green histogram in B and C) so that a flip of W^6.48^ back to state 1 (step IV) is required, arriving at the gray structure in G (one frame after the green structure in G). Further opening of TM6 is then possible (step V), yielding the structure in H. Finally, the G-protein may bind (step VI) to yield fully active Y1R (with W^6.48^ in state 1).

The exact structures and PCA locations can vary during the activation process. However, the key point is that W^6.48^ rotameric state 2 stabilizes conformations that should more readily bind NPY, and furthermore, when W^6.48^ is in state 2 in NPY-bound Y1R, the backbone structure is very close to structures accessible to apo Y1R, so that NPY may prefer to bind apo Y1R when it is in one of these structures. Interestingly, though, the backbone structures immediately before W^6.48^ flips to state 2 in apo Y1R and immediately after W^6.48^ flips back to state 1 in NPY-bound Y1R are almost identical to the backbone structures in state 2. Then, it is not a requirement for W^6.48^ to be in state 2 for NPY binding to occur. The presence of W^6.48^ state 2, however, increases the probability that Y1R is in a favorable backbone conformation for NPY binding. This has the potential to modify, or, “fine-tune” Y1R dynamics. In particular, we believe it is mostly likely to accelerate Y1R binding kinetics while having only a small impact on ligand binding affinity (suggesting that both NPY binding and unbinding is accelerated). This is further investigated with a simple kinetic model in the Supporting Information, section 5.

## Conclusions

Conserved motifs, such as the E­(D)­R^3.50^Y^12^ and the W^6.48^ “toggle switch”^20^ residues play critical roles in many GPCRs.^18^ For this study, the role of the W^6.48^ “toggle switch” of neuropeptide Y1 receptor on microstate regulation is investigated, where a tryptophan residue is indeed conserved in 6.48 position, but mutation studies (W276A)^37^ show that it is not required for G-protein signaling, raising the question as to the role it plays.

Recent research has established that GPCRs exist in a variety of states defined by their activation and deactivation or defined by their binding state (these are not always one-to-one; e.g., GPCRs with a large basal activity enter the active state without extra- or intracellular binding partners). Furthermore, for a given binding state, there may be considerable exchange between multiple microstates. If we consider the microstates observed via MD for apo Y1R (Figure 1), we note that some exhibit similarity to the structure of NPY or NPY/Gi bound Y1R, and so the relative populations of those microstates have the potential to influence the rate of ligand binding.

Then, presence of a tryptophan at the 6.48 position is not required for G-protein signaling,^37^ but it nonetheless can play a key role in regulating Y1R signaling behavior. Our investigation indicates that the rotameric state of W^6.48^ modulates the sampling of backbone microstates (Figure 6), which, in turn, can fine-tune the Y1R ligand-binding dynamics. A similar result was found for the β2-adrenergic receptor: mutation of R^3.50^ of the E­(D)­R^3.50^Y motif to alanine did not affect Gs signaling and receptor internalization, despite R^3.50^ being a key microswitch in the receptor.^52^ The proposed explanation is that R^3.50^ is a “crucial but balancing” microswitch, where the ionic lock stabilizes both the inactive and active states of the β2-adrenergic receptor, so that its mutation would similarly affect inactive and active states. This is similar to what is observed here: removal of a microswitch does not strongly affect ligand binding affinity, likely because its mutation affects on- and off rates to a similar extent (SI Figure 11 and SI Figure 2), but the microswitch may be able to increase binding rates by favoring certain microstates.

In Y1R, we would propose that evolutionary selection has not only placed the tryptophan at this key position but also chosen neighbors that provide the correct degree of energetic stabilization to each tryptophan rotameric state to correctly tune Y1R binding rates. Then, our results show that sampling a structurally diverse set of microstates is important for modulating GPCR function. Furthermore, side-chain rotameric states at structurally important positions, such as the 6.48 position immediately below the transmembrane-helix 6 kink, sometimes play key roles requiring a rotameric “toggling” to allow protein activation^20,31–34^ but may also play a more subtle by “fine-tuning” the binding rates by modulating microstate sampling.

## Supplementary Material



## Notes

Detailed explanations of sample preparations, experimental NMR details, setup of MD simulations, and additional figures (PDF)

## Abbreviations

GPCR: G-protein-coupled receptor
Y1R: neuropeptide Y receptor Y1
MD: molecular dynamics
NMR: nuclear magnetic resonance
NPY: neuropeptide-Y
PCA: principal component analysis
