# Supplementary material for: Fine-Tuning of the Neuropeptide Y1 G Protein-Coupled Receptor by the Tryptophan6.48 “Toggle Switch”
Source: J Am Chem Soc. 2025 Dec 19;148(1):194–205. doi: 10.1021/jacs.5c07143 (PMC12814323; doi:10.1021/jacs.5c07143)
Supplement: Supplementary file 1 [file ja5c07143_si_001.pdf]

Supporting information for

Fine-Tuning of the Neuropeptide Y1 G Protein-Coupled Receptor by the Tryptophan$^{6.48}$" Toggle Switch"

Matthias Voitel,[#,1] Maik Pankonin,[#,1] Alexander Vogel,[1] Karl Leitner,[2] Anette Kaiser,[2] Daniel Huster,[1] Peter W. Hildebrand,[1] Albert A. Smith*,[1]

[1]Institute for Medical Physics and Biophysics, Leipzig University, 04107 Leipzig, Germany

[2]Departement of Anesthesiology and Intensive Care, University of Leipzig Medical Center, 04103 Leipzig, Germany

[#] equal contributions

* albert.smith-penzel@medizin.uni-leipzig.de

1. SAMPLE PREPARATION 2

DNA CONSTRUCT PREPARATION 2
CELL FREE EXPRESSION AND PURIFICATION OF Y1R 2
RECEPTOR FOLDING 2
LIGANDS 3

2. EXPERIMENTAL MEASUREMENTS 3

FLUORESCENCE POLARIZATION ASSAY 3
IN CELL FUNCTIONALITY ASSAYS 3
FLUORESCENCE MICROSCOPY 3
IP1 ACCUMULATION ASSAY 4
ARRESTIN-BRET ASSAY 4
NMR MEASUREMENTS 4

3. MD SIMULATION 4

4. ADDITIONAL FIGURES 6

5. ENERGETIC MODELS FOR THE KINETIC AND THERMODYNAMIC EFFECTS OF MICROSTATE STABILIZATION 13

6. REFERENCES 17

1. Sample Preparation

DNA construct preparation

The gene of hY1R was cloned into a pIVEX2.3d vector with a modified NcoI site (ATG to GCG). Between the M1 and N2 an H-tag (KPYDGP)[1] was introduced and a Poly-6-His tag (PGGGSHHHHHH) was attached C-terminally (SI Figure 4). For in cell assays, the wt gene was cloned into an N1-vector, containing a C-terminally fused eYFP as described by Dinger et al.[2] Receptor variants were produced by mutation of one or more of the five tryptophans in Y1R into phenylalanine by site-directed mutagenesis.

To obtain enough DNA for cell free expression or transfection, already purified plasmids or PCR products from site-directed mutagenesis were transformed into *E.coli* XL1 blue, cultivated in lysogeny broth medium and purified with Mini or Midi plasmid preparation Kit (Macherey-Nagel). Sanger sequencing checked the sequence identity.

For IP1 accumulation, a chimeric G protein ($G\alpha_{\Delta 6qi4myr}$) was used to signal via phospholipase C pathway (G_q) while binding to Y_1R (G_i).[3] The construct is inserted into a pcDNAI-based expression plasmid.[4]

For Arrestin-recruitment assays, a nanoluciferase was genetically fused to the N-terminus of arrestin3 yielding a Nluc-arr3_pcDNA3 vector as described by Wolf et al.[5]

Cell free expression and purification of Y1R

Y1R was expressed in a cell-free approach. The protocol is adapted from Schwarz et al.[6] and was established for neuropeptide Y receptors by Krug et al.[7] This cell-free expression is based on an S12 extract from *E. coli*. For expression, a master mix was prepared containing HEPES buffer, magnesium and potassium acetate, polyethylenglycol 8000, sodium azide, calcium folinate, dithiothreitol, NTPs, protease inhibitor mix, potassium phosphoenolpyruvate, lithium potassium acetyl phosphate and all 20 amino acids. This was separated into two parts in the ratio of 16.05 to 0.95. Pyruvate kinase, tRNA, RiboLock, the DNA template and S12 extract were added to the smaller fraction forming the reaction mixture and transferred into a dialysis bag. This was placed into the remaining master mix supplemented with further amino acids and buffer components. The expression was performed at 34°C and 80 rpm for 24 h. The precipitated protein was solubilized in 50 mM NaP and 15 mM SDS buffer pH 6.0 with 50 mM DTT, which was removed subsequently by dialysis. After solubilization, the protein could be purified. The detailed protocol for extract preparation and cell free expression containing all relevant information is published in the SI of Krug et al.[7] Tryptophan with natural abundance isotopes was replaced by U-$^{13}C^{15}N$ tryptophan for receptor preparations used for NMR.

The purification of Y1R was performed via the His_6-tag over a HisTrap^{TM} FF crude 5 mL column (Cytiva) on an ÄKTA purifier 900 (Cytiva). The pH of the sample was adjusted to 7.8 before it was loaded onto the column. The used equilibration and elution buffer solutions contained 50 mM NaP and 15 mM SDS. The protein was eluted by a pH-shift from 7.8 to 4.5 and the purified receptor fraction was collected at a threshold of above 20 mAU UV absorption. Protein content was determined by UV-absorption at 280 nm wavelength with a NanoDrop Spectrophotometer ND-1000 (Peqlab Biotechnologie GmbH).

Receptor folding

Receptor folding was based on a protocol for the neuropeptide Y2 receptor[8] and adjusted to Y1R. In detail, the purified receptor at concentration of 0.5 mg/mL was dialyzed against degassed refolding buffer (50 mM NaP (pH 8.9), 2 mM SDS, 1 mM EDTA, 2 mM GSH and 1 mM GSSG) for ~66 h. After addition of preformed mixed DMPC/DHPC micelles in 50 mM NaP (pH 8) at a ratio of 1:200:800 (receptor:DMPC:DHPC) for membranes and 1:600:2400 for isotropic bicelles, a heat-ice cycling was

performed at 42°C and on ice for three rounds and 20 min per step. The deuterated DMPC-d_{54} was used for the preparation of NMR samples.

The formation of the isotropic bicelles was achieved by overnight dialysis against dialysis buffer (50mM NaP (pH 7), 1.5 mM DHPC) with 1 mM GSH, 0.5 mM GSSG and 1 mM EDTA. The receptor sample was concentrated by dialysis against dialysis buffer and 30 % PEG and two additional dialysis steps with dialysis buffer removed the remaining redox shuffling system. For fluorescence polarization assays, GPCRs in isotropic bicelles are used.

In order to obtain planar membranes for solid-state NMR,[8] instead of a dialysis after heat-ice-cycling, the folding for the production of receptor in membranes continued with addition of 50 mg/mL BioBeads SM-2 adsorbents (Bio-Rad) and shaking at room temperature overnight. Additionally, 50 mg/mL BioBeads were added twice followed by 2 h shaking until the sample turned turbid. After removal of the beads, the protein in membranes was pelleted by centrifugation at 4,000 x g and 4°C for 15 min. The pellet was washed three times with 50 mM NaP (pH 7) by resuspension followed by centrifugation. In the last round the protein content was measured by UV-absorption at 280 nm wavelength. The pellet could be stored at -20°C.

Ligands

pNPY and [Dpr^{22}(Atto520)]-pNPY were kindly provided by the service project Z03 of CRC1423.

2. Experimental Measurements

Fluorescence polarization assay

The receptor in isotropic bicelles was diluted serially in the concentration range between 10^{-5} and $10^{-10.5}$ M with 50 mM NaP (pH 7) containing 1.4 mM DHPC and 50 nM [Dpr^{22}(Atto520)]-pNPY was added. 100 µL were transferred into a COR96fc half area UV transparent 96-well plate (Corning) in triplicates. Samples were incubated for 2 h in the dark at 37°C. Fluorescence polarization was measured using TECAN Spark plate reader with excitation wavelength at 515/20 nm and emission wavelength at 560/20 nm. Assays were prepared with n ≥ 2 repetitions. Data was analyzed with the Software OriginPro (Version 2019) and fitted with the implemented "DoseResp" function with "p" set to 1. EC_{50} values are derive from the fit.

In cell functionality assays

Fluorescence Microscopy, inositol phosphate (IP1) accumulation assay and arrestin-BRET assay were performed in HEK293 cells as described before[9] to investigate if the mutation of tryptophan residues into phenylalanine in Y1R does interfere with receptor function.

In brief, cells were cultivated in Dulbecco's Modified Eagle Medium with Ham's F12 (Lonza) supplemented with 15 % fetal calf serum (Lonza) at 5% CO_2 and 37°C. Cells were transfected, re-seeded in a suitable plate, stimulated with NPY if needed and treated according to the assay protocol.

Fluorescence Microscopy

~160,000 cells per well have been seeded into an 8-well µ-slide (IBIDI) and incubated for 24 h. Transfection was performed using 1000 ng of Y1R-eYFP plasmid and Lipofectamin 2000 (Invitrogen) according to manufacturer's protocol in Gibco OptiMEM (Thermo Fisher Scientific). After 1 h of transfection, medium was exchanged and cells were incubated overnight. Medium was renewed again and nuclei were stained with 5 µg/mL Hoechst33342. Microscopy was performed on an Axiovert Observer Z1 microscope (Carl Zeiss) with ApoTome2 imaging system, enabling quasi-confocal imaging (filters: 46 for YFP and 02 for Hoechst33342 stain).

IP1 accumulation assay

70% confluent cells in six-well plates were transfected with Metafectene® Pro (Biontex) using 3200 ng Y1R_eYFP_N1 and 800 ng $G_{\Delta 6qi4myr}$ encoding plasmid. After 24 h of transfection, 20,000 cells were seeded per well in fresh medium into a white 384-well plate (Greiner Bio-one). Medium was removed after overnight incubation and cells were treated according to the manufacturer's protocol of the HRTF® assay kit (CisBio). In particular, cells were stimulated with different NPY concentrations in HBSS + 20 mM LiCl for 1.5 h. 3 µL each of IP1-d2 and Ab-Cryptate were added, incubated for 1h at RT under gentle agitation, and fluorescence was measured (TECAN Spark; excitation wavelength: 320 nm/25 nm, mirror: Dichroic 510, emission wavelength: 620 nm/10 nm (FRET-donor); 665 nm/8 nm (FRET-acceptor)). Data was analyzed using Prism/Graphpad (Version 10.3.0) using the built-in three-parameter logistic fit.

Arrestin-BRET assay

For the arrestin-BRET assay, 60 ng Nluc-arrestin3 plasmid, and 3940 ng Y1R-eYFP_N1 were transfected with Metafectene® Pro (Biontex) into cells at 70% confluence in six-well plates for 24 h. We ensured by DNA titration that these conditions saturate the Nluc-arr3 donor, and hence the maximal BRET signal and EC_{50} depend only on the functionality of the receptor construct, not the stoichiometry of the receptor caused by variable transient transfection. After exchange of the medium, cells were seeded into poly-D-lysine coated 96-well plates (133,000 cells per well) and incubated overnight. Medium was removed and 100 µL of BRET buffer (HBSS + 25 mM HEPES, pH 7.4) was added followed by 50 µL of coelenterazine h (16.7 µM in BRET buffer) and 3 min incubation at 37°C. Cells were stimulated by addition of 50 µL NPY in a concentration range between 10^{-5} and 10^{-10} M in BRET buffer. 6 and 11 min after addition of NPY luminescence was measured at 400 – 470 nm and fluorescence was measured at 535 – 650 nm (TECAN Spark). Data was analyzed using Prism/Graphpad (Version 10.3.0).

NMR measurements

The $^{13}C^{15}N$ tryptophan labeled receptor pellet in DMPC membranes was resuspended in 900 µL 50 mM NaP buffer (pH 7) containing pNPY in ratio 2:1 (ligand:receptor) if necessary. After overnight incubation at 4 °C under agitation, the sample was transferred into a 3.2 mm NMR MAS rotor (Bruker) using ultracentrifugation at 30,000 x g for 1.5 h at 4°C. $^{13}C^{13}C$-DARR[10] correlation experiments (10 ms mixing) were performed at Bruker Avance Neo 700 MHz NMR spectrometer under 12 kHz MAS at –30°C and analyzed with TopSpin 4.0.9 (Bruker). For DARR spectra, 2880–2944 scans were accumulated with 80 t_1 and 640 t_2 increments were acquired, for acquisition times of 9.34 ms and 1.11 ms, respectively. Time data was processed with SINE (indirect) or QSINE (direct) window functions with a sine bell shift of 2.

3. MD Simulation

Four different MD setups were performed, for the unbound (apo) receptor, for NPY-bound receptor, for NPY and Gi bound receptor, and for antagonist-bound (UR-MK299) receptor. Each setup was then run 3 times with different initial velocities. The NPY/Gi-bound receptor simulation was initialized from the cryo-EM structure of Tang et al. (PDB: 7X9A), [11] and the NPY-bound receptor simulation was setup by removing Gi from this structure. Simulations of Y1R in its apo and inactive (antagonist bound) states followed the same procedure as Vogel et al.,[12] starting from the antagonist-bound crystal structure (PDB: 5ZBQ).[13] Due to the N- and C-termini being unresolved in the pdb files, the residues N-terminally (M1-A27) and C-terminally (F337-I384) are missing in the final receptor structure used for the MD simulation. The stabilizing palmitoylation site Cys338, missing in the Cryo-structure, was introduced manually using PYMOL (version 2.5.2, Schrödinger LLC) in preparation of the MD Simulation. Unresolved residues of the proteins of the Gi complex were introduced via homology modeling using the PDB structure 6OT0[14] as a template. Artificial N-Termini were acetylated, artificial C-Termini methylated to introduce a bulky, neutral cap of the terminus unable to form unwanted hydrogen bonds. Other termini were capped using the standard charged natural protein terminus for a pH of 7, unless they had to be adjusted due to known

posttranslational modifications. Table 1 gives an overview of all residues either truncated or modeled via homology and the capping of the termini for each setup.

Table 1: Overview of the terminus setup chosen in preparation of the simulations. Capping (methylation, acetylation and standard) were done using provided capping option in CHARMM-GUI. "standard" capping refers to the protonation state mostly likely encountered for the N-/C-Terminus at a pH of 7.

protein	N-TER	C-TER
**Y1R**	M1-A27 truncated, acetylated	F340-K383 truncated, methylated
**NPY**	standard	amidated
**Gi alpha**	homology model (G2-L5), standard (Gly)	standard
**Gi beta**	homology model (S2-L4), acetylated	standard
**Gi gamma**	homology model (N2-S5), acetylated	methylated

Additionally, the α-helical domain (I56-T181) and short loop (L232-E239) of the Gi-alpha subunit were not resolved in the cryo-EM structure, and therefore were homology modeled based on the resolved Gi-alpha subunit of the human Smoothened-Gi complex (PDB: 6OT0[14]) as a template.

The receptor cavities were filled with water molecules using *dowser*.[15] The amino acids were kept in their standard protonation state of the charmm36 force field, with exception of the Y1 receptor residues Asp2.50 and Glu3.49, which were protonated to emulate the active state of the receptor in the agonist-bound setups.[16,17] Likewise, Asp2.50 was protonated in the antagonist-bound state of Y1R but was deprotonated in preparation for the apo state, with a Na^+ ion positioned in close proximity (described in more detail in Vogel et al.).[12] As intended Cys338 was palmitoylated using the CYSP path of the CHARMM force field. The receptor without the Gi complex was embedded in a POPC bilayer with 101 molecules in the upper and lower leaflet or 211 molecules for the upper and 204 molecules for the lower leaflet in the setup including the G protein complex respectively. The embedded receptor with Gi was inserted into larger simulation boxes with the x-y-z dimensions of 125-125-181 Å which afterwards was filled ~64000 TIP3 water and seven sodium ions (to neutralize the system) as reported previously. Similarly, the system without the Gi complex present was inserted into a 90-90-130 Å box and filled with ~22000 TIP3 water and seven chloride ions.

Simulations were run at 310.15 K, with a pressure of 1.013 Bar, using an NPT ensemble in GROMACS 2021.2, with the CHARMM36 force field.[18] Application of the LINCS algorithm[19] allowed a timestep of 2 fs. NPY/Gi-bound Y1R simulations contained about 358000 atoms, whereas NPY-bound contained about 98000 atoms. Energy minimization was performed with the steepest descents algorithm, with a threshold of 1000 $kJ{*}mol^{-1}{*}nm^{-1}$. Seven steps were used for equilibration with decreasing restraints of the protein sidechains, backbone, and on POPC molecules. Table 2 provides additional details of the equilibration step. A total of 3 independent MD simulations for each setup were run for at least 10 µs each (apo: 26.3, 26.3, 15.5 µs, NPY-bound: 16.7, 17.0, 16.9 µs, UR-MK299 bound: 22.0, 21.8, 21.8 µs, NPY/Gi-bound: 10.5, 10.7, 11.0 µs).

Table 2: Equilibration steps used to adjust the Y1R model for the charmm36 forcefield. BB restraints: flexible, positional restraints of protein backbone; SC restraints: flexible, positional restraints of protein side chains; Lipid restraints: flexible, positional restraints for POPC molecules.

**Step**	**Step 1**	**Step 2**	**Step 3**	**Step 4**	**Step 5**	**Step 6**
**Duration**	125 ps	125 ps	125 ps	250 ps	250 ps	250 ps
**BB restraint**	4000	2000	1000	500	200	50
**SC restraint**	2000	1000	500	200	50	0
**Lipid restraint**	1000	400	400	200	40	0

The simulations were analyzed using the software *GROMACS,*[20,21] *VMD*[22] and the inhouse python tools *mdciao* and *pyDR.* Visualization of the simulated trajectories was done using *VMD* and *chimeraX*[23].

4. Additional Figures

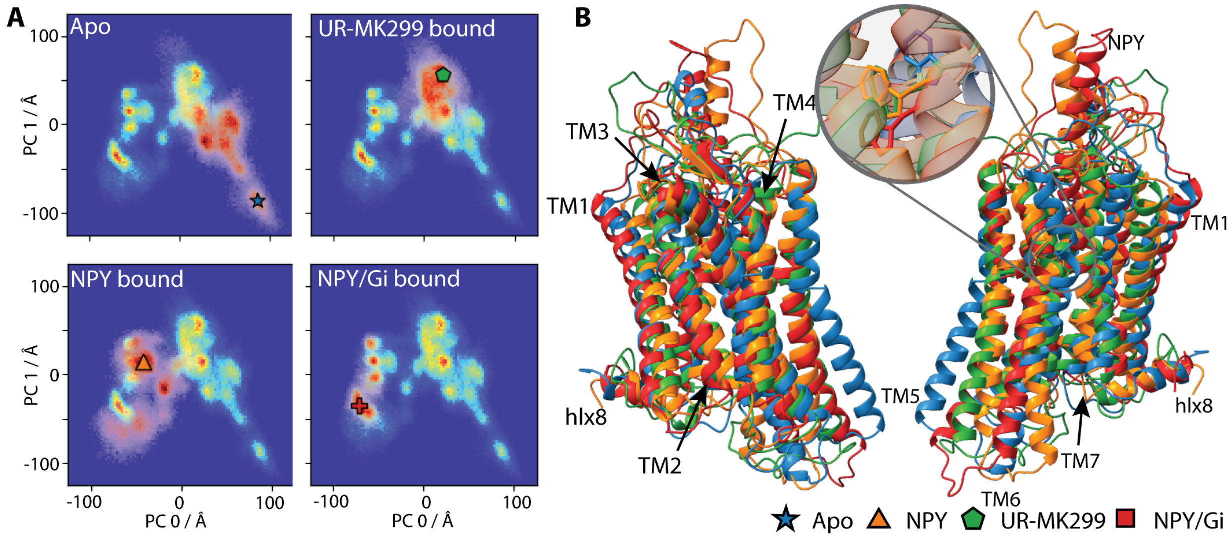

SI Figure 1. PCA of all trajectories. **A** overlays the combined PCA of all trajectories (rainbow), with the contribution to that PCA from one state of Y1 (apo, NPY-, NPY/Gi-, and UR-MK299-bound) overlayed in red. For each plot, one point is selected, corresponding to a representative structure shown in **B.**

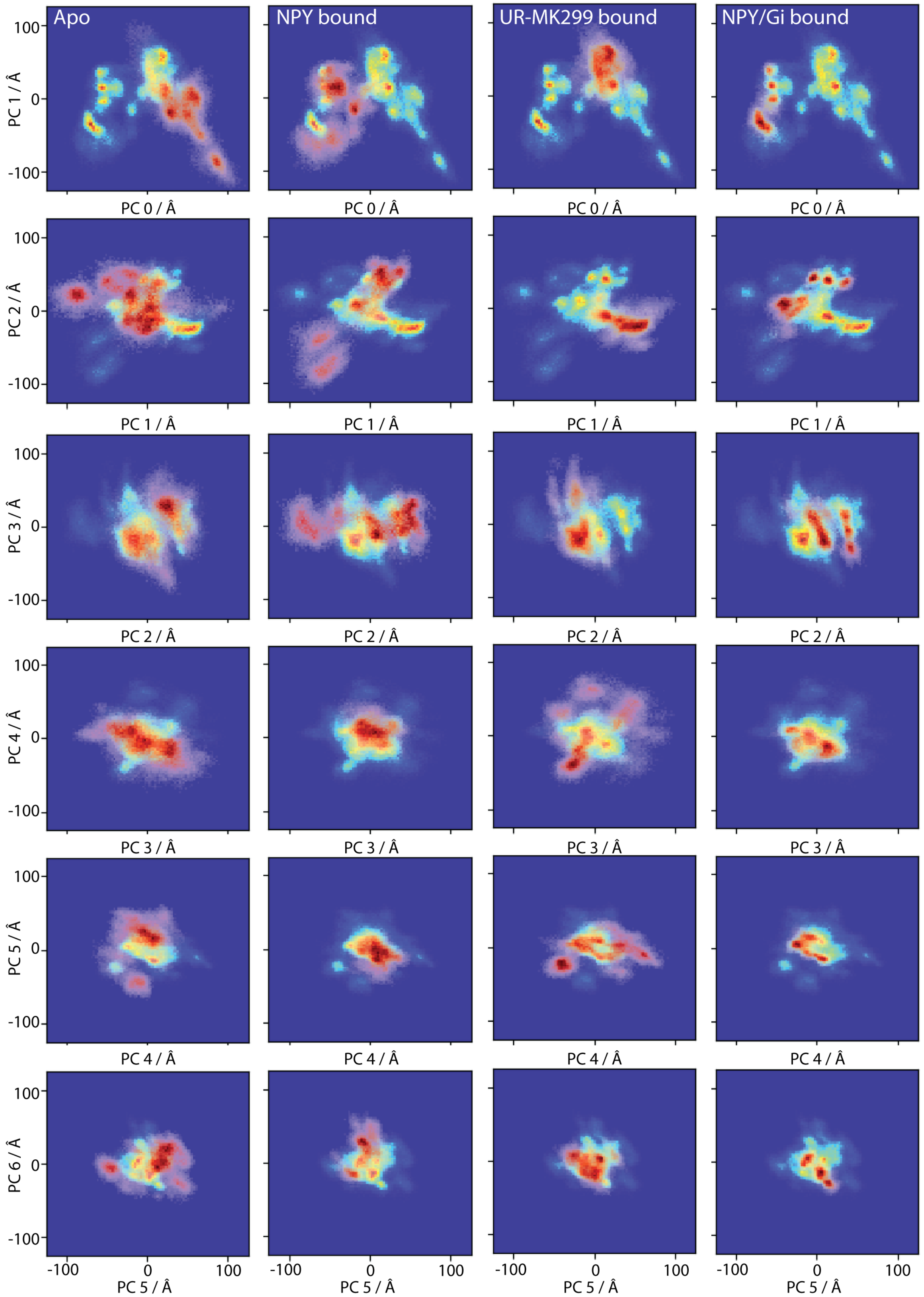

SI Figure 2. PCA histograms showing the first seven (0–6) principal components. Each row is a different pairing of principal components (PC 0 vs. PC1, PC 1 vs. PC2, etc.). Rainbow histograms indicate the full PCA of all trajectories, whereas each column corresponds to a different set of trajectories (apo, NPY-bound, UR-MK299-bound, NPY/Gi bound); the overlaid red histogram shows contributions from that set of trajectories to the total PCA.

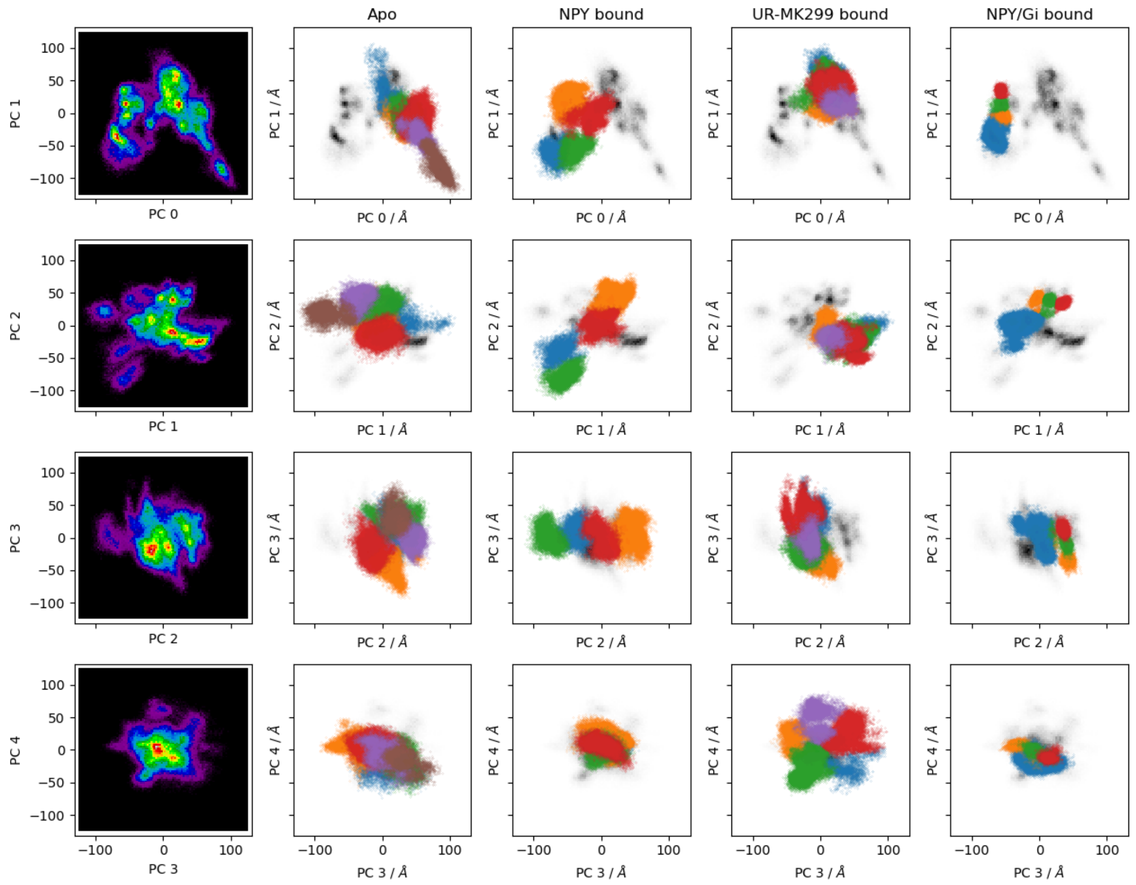

SI Figure 3. PCA clustering. Each row corresponds to a different pairing of principal components. The leftmost plots are the total PCA, whereas each following column shows how each of the four states of Y1 were clustered. Apo Y1 is grouped with 6 clusters based on PC0-PC3, NPY-bound with 4 based on PC0-PC2, UR-MK-299 with 5 clusters based on PC0-PC4, and NPY/Gi-bound with four clusters based on PC1-PC2.

```
         10         20         30         40         50         60
MKPYDGPNST LFSQVENHSV HSNFSEKNAQ LLAFENDDCH LPLAMIFTLA LAYGAVIILG
         70         80         90        100        110        120
VSGNLALIII ILKQKEMRNV TNILIVNLSF SDLLVAIMCL PFTFVYTLMD HWVFGEAMCK
        130        140        150        160        170        180
LNPFVQCVSI TVSIFSLVLI AVERHQLIIN PRGWRPNNRH AYVGIAVIWV LAVASSLPFL
        190        200        210        220        230        240
IYQVMTDEPF QNVTLDAYKD KYVCFDQFPS DSHRLSYTTL LLVLQYFGPL CFIFICYFKI
        250        260        270        280        290        300
YIRLKRRNNM MDKMRDNKYR SSETKRINIM LLSIVVAFAV CWLPLTIFNT VFDWNHQIIA
        310        320        330        340        350        360
TCNHNLLFLL CHLTAMISTC VNPIFYGFLN KNFQRDLQFF FNFCDFRSRD DDYETIAMST
        370        380        390        400
MHTDVSKTSL KQASPVAFKK INNNDDNEKI PGGGSHHHHH H
```

SI Figure 4. Modified amino acid sequence for the Y1R construct used in this study. Changes relative to the wildtype sequence are marked. Gray: C-terminal His6 purification tag with PGGGS linker, Cyan: H-tag for expression enhancement,[1] yellow: five tryptophans which were mutated into phenylalanine, with location and number of mutations depending on the sample.

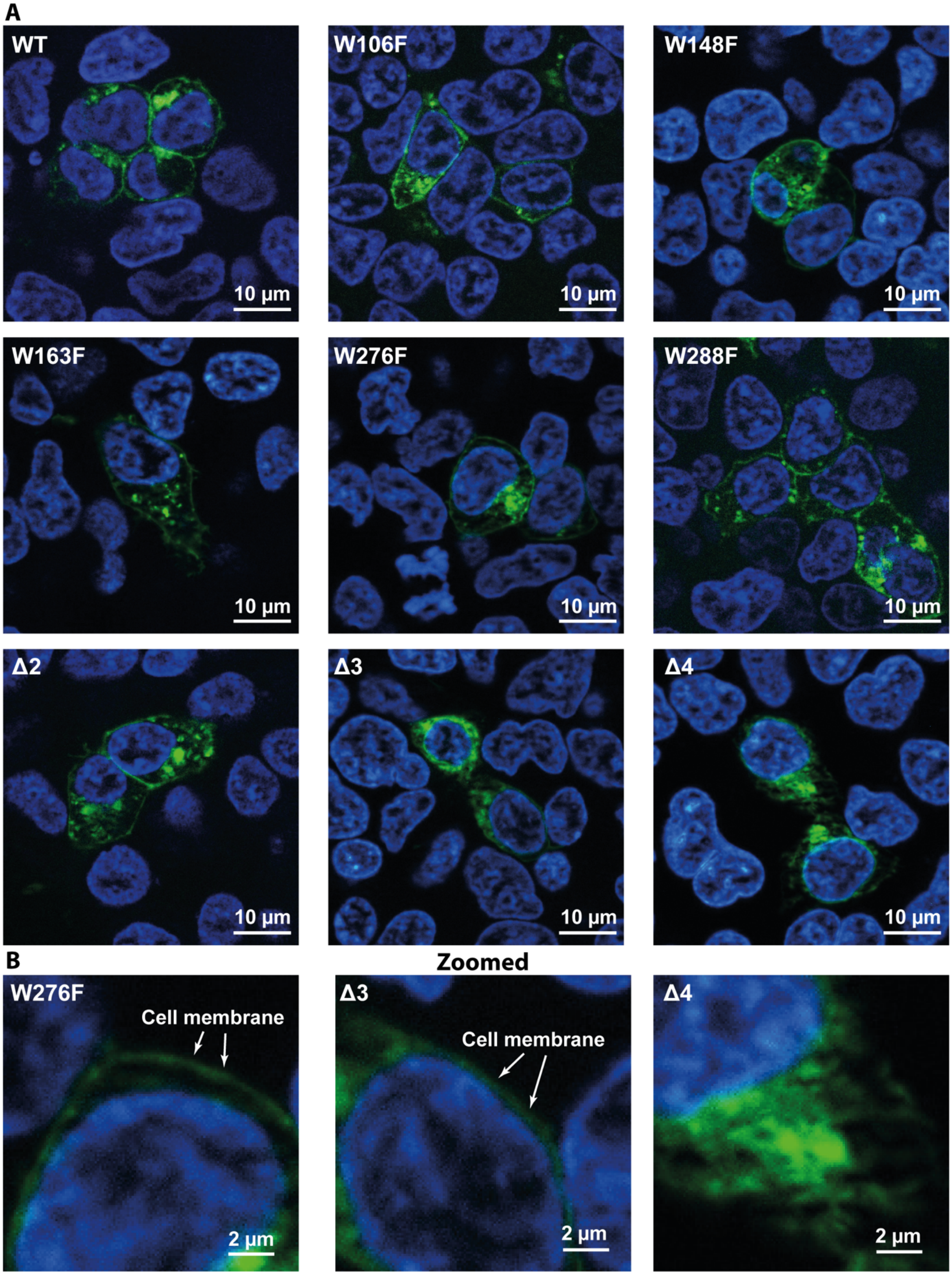

SI Figure 5. Fluorescence microscopy images of HEK293 cells transiently transfected with Y1R variants fused to eYFP. Green shows expressed receptor and its localization within the cells. The nuclei are stained with Hoechst 33342 in blue. Representative images from n ≥ 4 repetitions. **A** shows the full image and **B** zooms in on selected images, to highlight the visibility of Y1R in the cell membrane for W276F and Δ3. In Δ4, we do not clearly see Y1R in the cell membrane

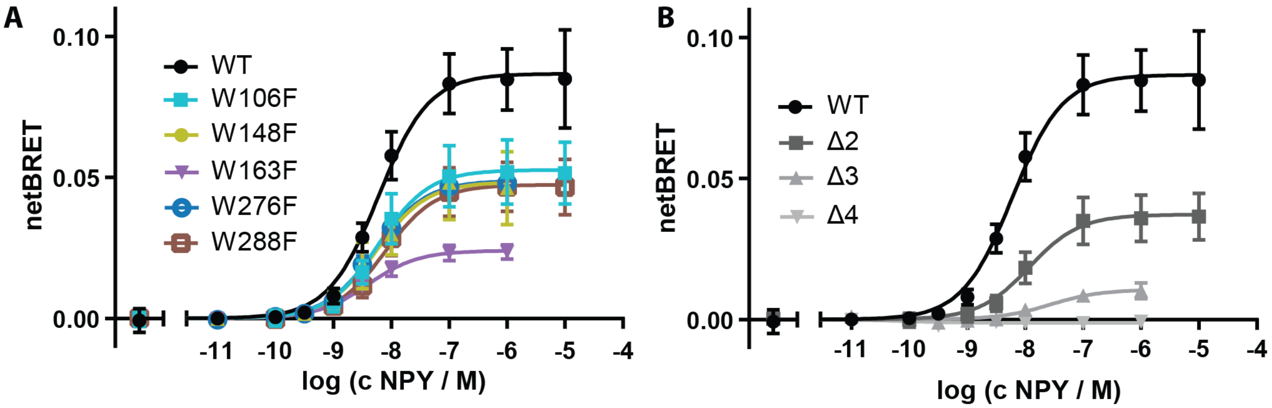

SI Figure 6. Arrestin BRET assays of tryptophan deficient Y1R mutants in HEK293 cells. **A** and **B** show the BRET signal reporting on arrestin binding. **A** shows the effect of individual mutations of tryptophan to phenylalanine for all five tryptophans (W106F cyan, W148F olive, W163F magenta, W276F blue, W288F brown) compared to the WT receptor (black). **B** shows the WT (black, circles) and multiple mutants Δ2 (W106F,W288F, squares, dark gray), Δ3 (W106F,W148F,W288F, triangles, medium gray), Δ4 (W106F,W148F,W163F,W288F, inverted triangles, light grey). Data are the mean with error bars of one standard deviation of three independent biological assays conducted in technical triplicate.

Table 3: logEC_{50} values for binding and functional assays

**NPY-binding log**EC_{50}		**IP1 accumulation log**EC_{50}		**Arrestin BRET log**EC_{50}	
**WT**	-6.18 ± 0.09	**WT**	-8.76 ± 0.08	**WT**	-8.22 ± 0.11
**Δ3-Trp**	-6.18 ± 0.04	**W106F**	-8.77 ± 0.26	**W106F**	-8.22 ± 0.18
**Δ4-Trp**	-6.20 ± 0.23	**W148F**	-8.59 ± 0.13	**W148F**	-8.25 ± 0.20
		**W163F**	-8.61 ± 0.14	**W163F**	-8.40 ± 0.09
		**W276F**	-8.64 ± 0.13	**W276F**	-8.27 ± 0.05
		**W288F**	-8.58 ± 0.15	**W288F**	-8.10 ± 0.17
		**Δ2-Trp**	-8.34 ± 0.24	**Δ2-Trp**	-7.93 ± 0.20
		**Δ3-Trp**	-8.09 ± 0.13	**Δ3-Trp**	-7.56 ± 0.29
		**Δ4-Trp**	n.d.	**Δ4-Trp**	n.d.

n.d. not detectable

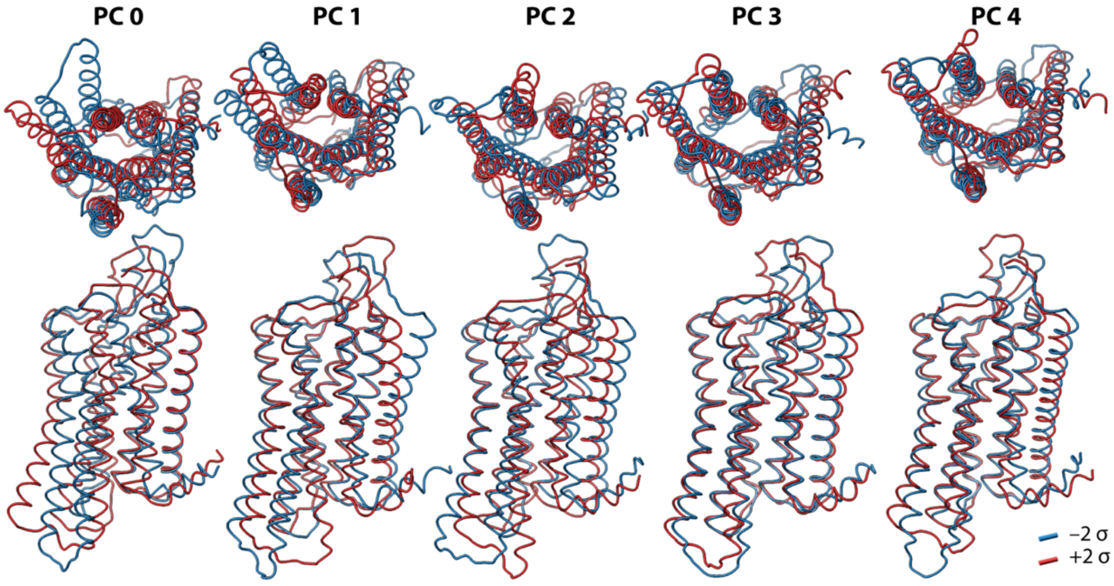

SI Figure 7. Deviation of the first 5 PCs from the mean Y1 structure. Each column plots two view of the Y1 structure, +2 (red) or -2 (blue) standard deviations away from the mean Y1 structure.

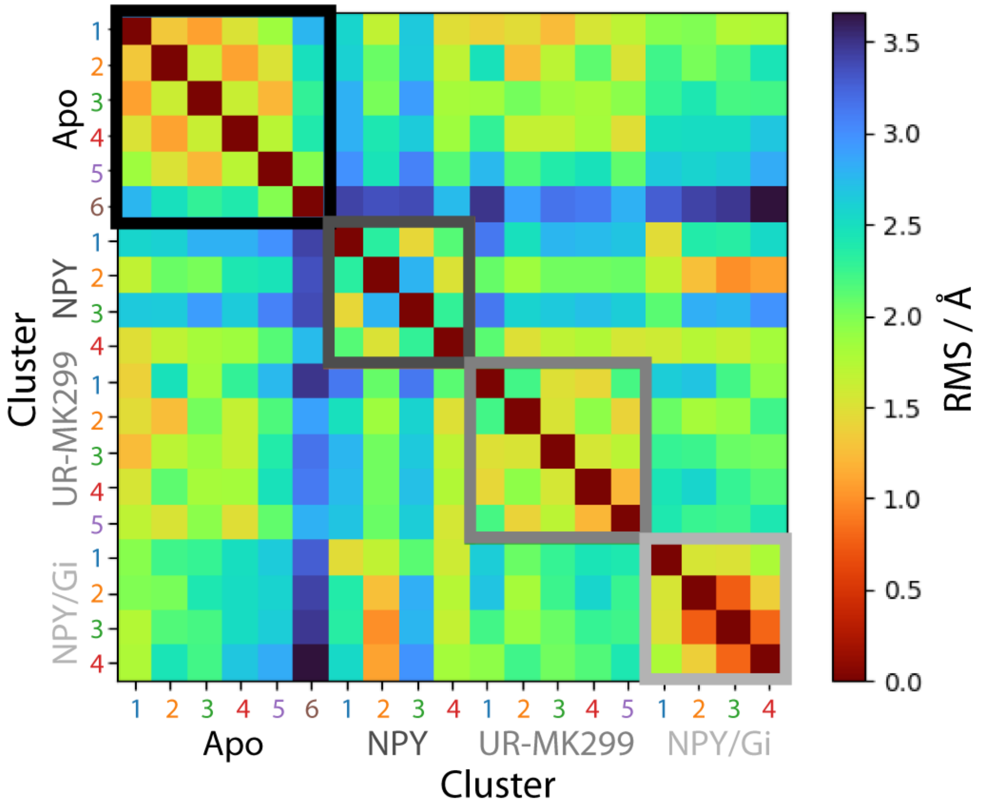

SI Figure 8. PCA cluster RMS. Each square compares the RMS between the mean structures for two clusters, where similar clusters tend towards dark red, and different clusters tend toward dark blue (colorbar to the right). Clusters are indicated in SI Figure 3, where color coding between the PCA plots and the axis labels in this figure indicate the corresponding clusters.

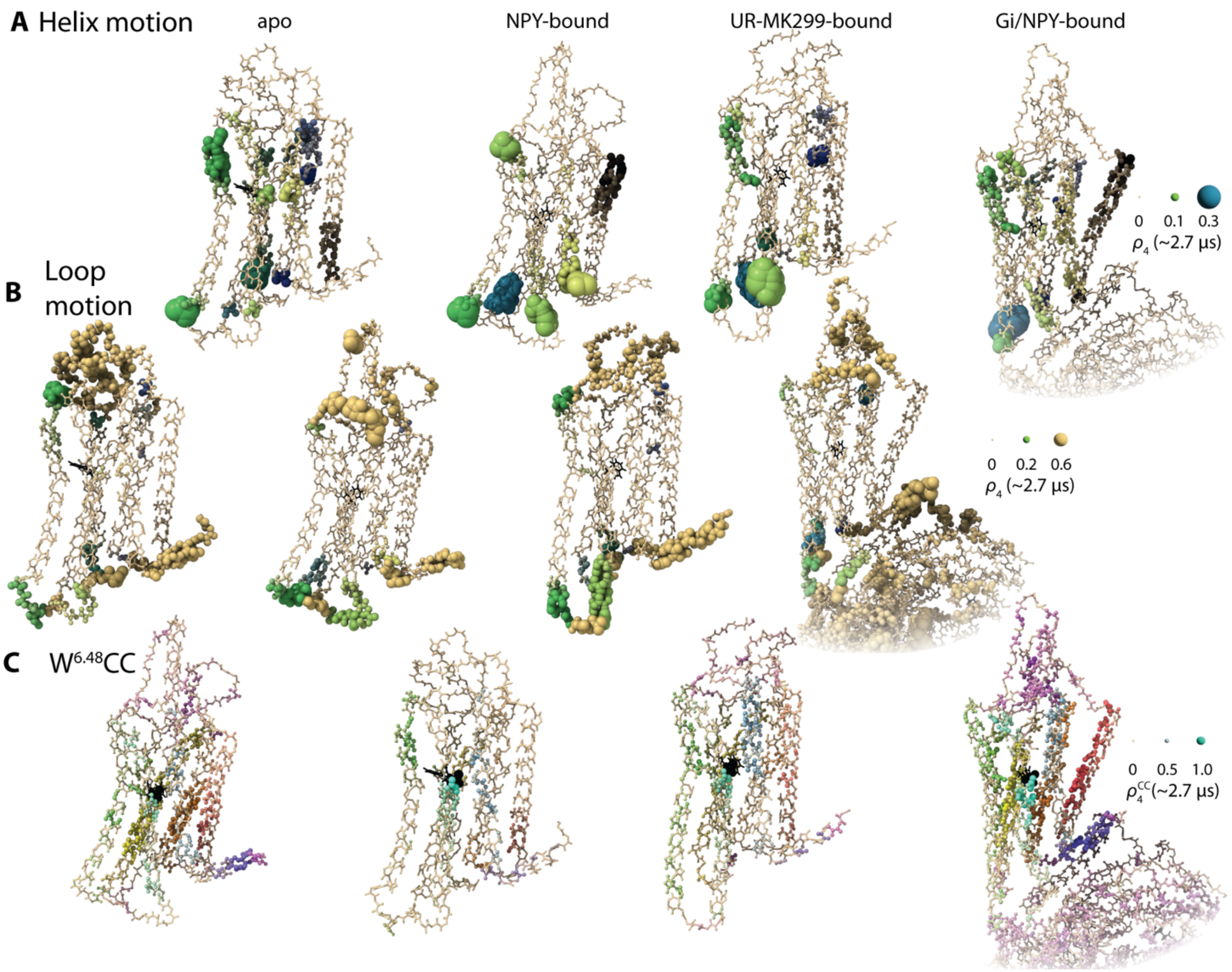

SI Figure 9. Dynamics comparison for each trajectory via detector analysis. 10 µs non-overlapping chunks (10000 frames) of the three trajectories for each binding state were analyzed using detector analysis (5 non-overlapping chunks for apo, 6 chunks for UR-MK299-bound, 3 chunks for NPY-bound, 3 chunks for NPY/Gi-bound). 6 detectors were used for analysis, where we show the slowest motion here, with the ρ_4 detector centered around 2.7 µs, with a width of 1.5 orders of magnitude (~450 ns to 16 µs). In **A** and **B**, the amplitude of backbone H–N reorientational dynamics is encoded into the atom radii and color intensity of the corresponding peptide plane (different colors are used for each helix to improve visibility. In **A**, extra- and intracellular loop residues and helix-8 are excluded to allow a better view of dynamics in the transmembrane helices, whereas in **B**, all residues are shown and we mainly see the larger-amplitude loop motions. **C** shows cross-correlation between the H–N bond of W276$^{6.48}$ (shown in black) and other H–N bonds. Correlation coefficients range between 0 and 1, and are encoded in the radii and color intensity of the corresponding peptide plane (as in **A**, **B**, each helix uses a different color for better visibility). Scales are shown to the right of each section.

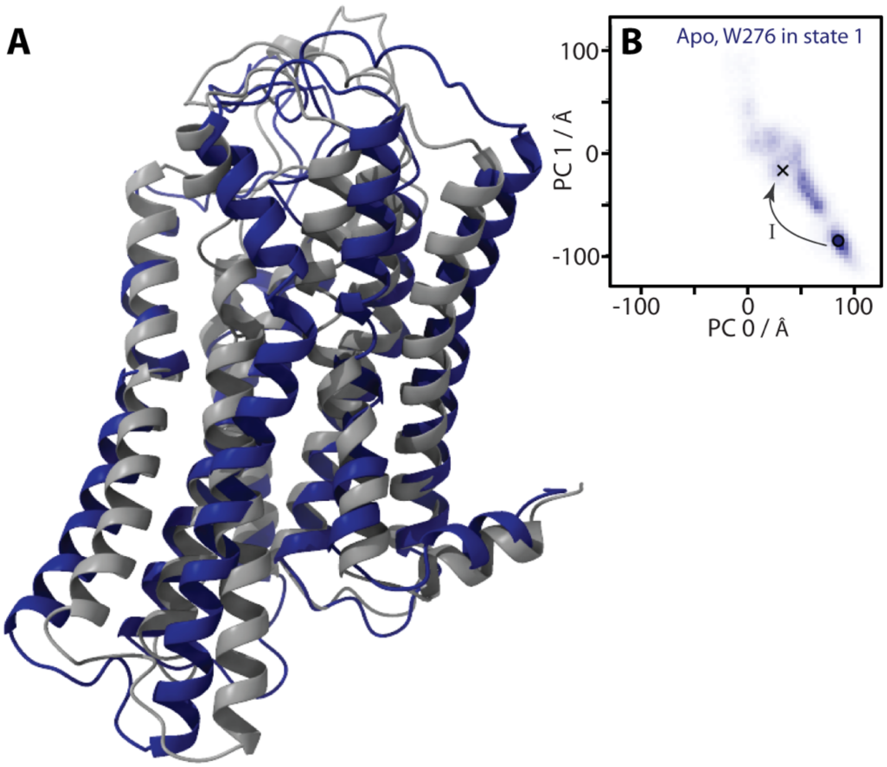

SI Figure 10. Overlay of Y1R apo structures with $W^{6.48}$ in rotameric state 1 from main text Figure 7. In **A**, the dark blue structure is the same as the dark blue structure in Figure 7E, and the grey structure corresponds to the grey structure in Figure 7F. Both structures correspond to apo Y1R with $W^{6.48}$ in state 1, but with different degrees of TM6 opening. The dark blue structure corresponds to TM6 closure, with high values of PC 0, whereas the grey structure has lower PC0 values corresponding to greater TM6 opening. The corresponding PCA is shown in **B**, where the histogram corresponds only to backbone structures observed with $W^{6.48}$ is in state 1.

5. Energetic models for the kinetic and thermodynamic effects of microstate stabilization

We have proposed that $W^{6.48}$ stabilizes apo Y1R microstates that are more similar to NPY-bound Y1R structures. This should modify binding rates without significantly modifying the receptor activity, however, it is not clear exactly how this should manifest. Therefore, in this section, we build a simple energetic model for observing what effects microstate stabilization might have.

We start with a review of the two-state model (apo and bound), but will then add "microstates", which in the model will simply split the apo and bound states into two additional states each (four states total). For the two-state model, it is well-established that populations of the two states are given by the difference in free energy of the two states:

$$\frac{p_a}{p_b} = \exp\left(-\Delta G_{a,b}/kT\right) = \exp\left(-\Delta H_{a,b}/kT\right)\exp\left(\Delta S_{a,b}/k\right) \tag{1}$$

Furthermore, we can consider the forward and reverse transfer rate constants ($k_{a,b}$, $k_{b,a}$) which are governed by the Arrhenius law, yielding

$$k_{a,b} = A_{a,b}\exp\left(-E_a^{a,b}\right) \tag{2}$$

$$k_{b,a} = A_{b,a} \exp\left(-E_a^{b,a}\right)$$

Since detailed balance requires that $p_a/p_b = k_{b,a}/k_{a,b}$ at thermal equilibrium, we may combine these equations to obtain

$$\Delta H_{a,b} = E_a^{b,a} - E_a^{a,b}$$
$$\Delta S_{a,b} = k_B \log\left(\frac{A_{b,a}}{A_{a,b}}\right) \tag{3}$$

However, if we want to consider the effect of stabilizing certain microstates, then the apo and bound states need to be split into at least two microstates (we label them I and II). Then, we will assume that a transition from apo microstate I will arrive in bound microstate I, and apo microstate II will arrive in bound microstate II, via different transition states. In this model, the total rate constant for apo to bound and vice versa is the average of rate constants from the two microstates. That is,

$$k_{a,b} = p_a^I k_{a,b}^I + p_a^{II} k_{a,b}^{II}$$
$$k_{b,a} = p_b^I k_{b,a}^I + p_b^{II} k_{b,a}^{II}, \tag{4}$$

where $k_{a,b}$ is the total rate constant from apo to bound, and $k_{a,b}^I$ is the rate constant when in microstate I and $k_{a,b}^{II}$ is the rate constant when in microstate II. In order to obtain simpler notation, we have defined p_a^I and p_a^{II} to add up to 1 (rather than to p_a). In this case, the total free energy of either the apo or bound state depends on the average of the free energy of the microstates minus an entropic term resulting from the presence of two microstates (labeled $T\Delta S_{I,II}$ below). We give the formula for the apo state below (the bound formula has different subscripts).

$$\Delta G_a = p_a^I \Delta G_a^I + p_a^{II} \Delta G_a^{II} - \underbrace{k_B T(p_a^I \log(p_a^I) + p_a^{II} \log(p_a^{II}))}_{T\Delta S_{I,II}} \tag{5}$$

SI Figure 11 shows an example of a system with exchange between apo and bound states, where apo and bound states are further separated into microstates I and II. In the figure, we show populations of apo and bound states (bottom), as well as populations of the individual microstates. We also show rate constants for transitions between apo and bound for each microstate, calculated from the difference in free energy of the individual microstates and the transition states (TS I, TS 2). Free energy calculated from eq. (5) is shown at the bottom of the plot (ΔG_a, ΔG_b, where $\exp(-(\Delta G_a - \Delta G_b)/kT) = p_a/p_b$ corresponds to the populations), and averaged transition rate constants are given at the top of the plot. Each microstate then has a different transition state free energy, resulting in 20x faster transitions from apo to bound for microstate 2. Note that most values given are taken just for example, with the exception that the ratio of microstates is taken from the ratio of $W^{6.48}$ rotameric state 1 vs. rotameric state 2 observed for apo and NPY-bound MD trajectories. The relative populations of apo and bound Y1R would result from 1.75 nM initial concentrations of NPY and Y1R, based on K_d=0.38 nM[24] $(0.37 * (1.75\ \text{nM}))^\wedge 2/(0.63 * (1.75\ \text{nM})) = 0.38\ \text{nM})$.

The model is intended to represent the case that apo microstate II in the model is stabilized by rotameric state II of $W^{6.48}$, where we assume a lower energy transition state due to structural similarity of the apo Y1R backbone to NPY-bound Y1R, and therefore obtain a faster rate constant for NPY-binding.

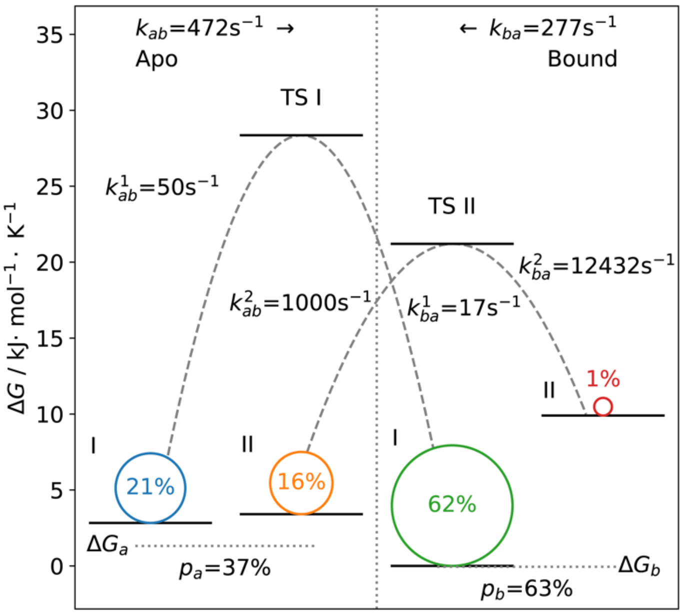

SI Figure 11. Proposed kinetic and thermodynamic model of ligand binding in the presence of two microstates (I, II) for both apo and bound Y1R. Apo and bound forms are separated from left to right, and each of these is further separated into two "microstates", I and II. In between apo and bound microstate I is the transition state I (TS I), and similarly for microstate II, we have TS II. For each microstate, a colored circle indicates its equilibrium population. The free energy of apo and bound resulting from the microstates is also shown ($\boldsymbol{\Delta G_a}$, $\boldsymbol{\Delta G_b}$), with the total apo/bound population shown below. Forward and reverse rate constants are indicated for microstates I and II ($\boldsymbol{k^I_{ab}}$, $\boldsymbol{k^{II}_{ab}}$, $\boldsymbol{k^I_{ba}}$ $\boldsymbol{k^{II}_{ba}}$), and the averaged forward and reverse rate constants are indicated at the top ($\boldsymbol{k_{ab}}$, $\boldsymbol{k_{ba}}$). The ratios of microstates I and II are taken from the ratios of $W^{6.48}$ state I and II (see main text Figure 4B), but ratios of apo to bound are chosen simply to keep populations within a similar range for all states. Rate constants are similarly arbitrary, where microstate II is chosen to result in 20x faster apo-bound transitions than microstate II.

With this energetic model, we can then investigate how stabilization of a microstate by the $W^{6.48}$ rotameric state could influence the overall kinetics and thermodynamics of the system. We postulate mutation of $W^{6.48}$ would result in a destabilization of apo microstate II. We note that if such a destabilization results in a large population shift, it would be inconsistent with investigations of $W276^{6.48}$A mutants, where only a small reduction in signaling activity was observed for the mutant relative to wild-type Y1R.[13] On the other hand, a change in ligand binding rate constants could be functionally important, but would not necessarily have a large effect on the ligand binding affinity or receptor activity.

Therefore, we destabilize apo microstate II relative to microstate I, since microstate II yields faster transitions from apo to bound. Specifically, we increase ΔG^{II}_a by 4.75 kJ/(mol*K) and decrease ΔG^{I}_a by the same amount, with the result shown in SI Figure 12. We also adjust the transition state free energies by half these amounts; the transition state structure should be similar to both the initial and final structures, so this should be a reasonable estimate of the influence of stabilization on the transition states. By making these adjustments, we find that the binding rate constant is decreased by nearly a factor of 6.8, representing a significant change in the binding rate.

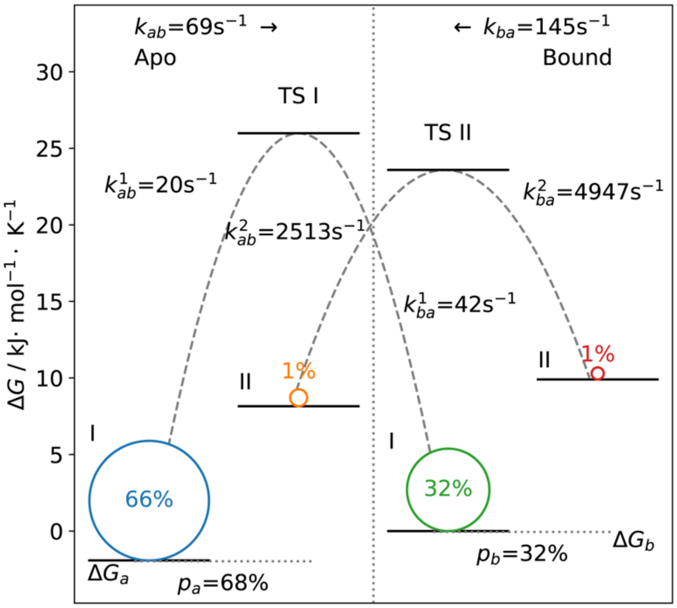

SI Figure 12. Effect of destabilizing apo state 2. Conditions from SI Figure 11 are taken here, along with adjustment of the energy of $\boldsymbol{\Delta G_a^I}$ by -4.75 kJ/(mol*K) and $\boldsymbol{\Delta G_a^{II}}$ by +4.75 kJ/(mol*K). Transition state energies of TS I and TS II are also adjusted, by -2.375 kJ/(mol*K) and +2.375 kJ/(mol*K), respectively. The result is a decrease in reaction rate constants

The apo vs. bound populations have also been modified; based on our initial concentration (1.75 nM), this would represent a change of the K_d from 0.38 nM to 2.5 nM. Theoretical ligand binding titrations are shown in SI Figure 13, for a fixed ligand concentration of 2.6 nM and varying receptor concentration. The change in EC_{50} values of 1.4 nM matches the change observed for the W276$^{6.48}$A mutant investigated by Yang et al.[13] Note that the value of $\Delta(\Delta G_a^{II}) = -\Delta(\Delta G_a^{I})$=4.75 kJ/(mol*K) was chosen to produce this agreement, but highlights the fact that despite the relatively low change in binding affinity with the W276$^{6.48}$A mutant, we may nonetheless obtain significant changes in binding rates, demonstrating that our postulate of "fine-tuning" the receptor behavior via modifying the binding rates without significant changes to the affinity is kinetically and thermodynamically reasonable.

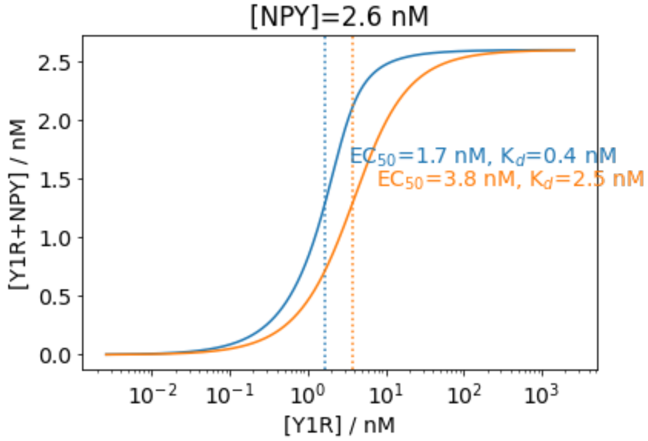

SI Figure 13. Theoretical ligand binding titrations for the energy diagrams in SI Figure 11 (blue) and SI Figure 12 (orange), where $\boldsymbol{K_d}$ is calculated based on the populations from each figure and an initial ligand and receptor concentration of 1.75 nM. The titration curves are based on a fixed ligand concentration of 2.6 nM, chosen to reproduce the EC_{50} values observed by Yang et al.[13]

6. References

(1) Haberstock, S.; Roos, C.; Hoevels, Y.; Dötsch, V.; Schnapp, G.; Pautsch, A.; Bernhard, F. A Systematic Approach to Increase the Efficiency of Membrane Protein Production in Cell-Free Expression Systems. *Protein Expr Purif* **2012**, *82* (2), 308–316. https://doi.org/10.1016/j.pep.2012.01.018.

(2) Dinger, M. C.; Bader, J. E.; Kobor, A. D.; Kretzschmar, A. K.; Beck-Sickinger, A. G. Homodimerization of Neuropeptide y Receptors Investigated by Fluorescence Resonance Energy Transfer in Living Cells. *J Biol Chem* **2003**, *278* (12), 10562–10571. https://doi.org/10.1074/jbc.M205747200.

(3) Kostenis, E. POTENTIATION OF GPCR-SIGNALING VIA MEMBRANE TARGETING OF G PROTEIN α SUBUNITS. *Journal of Receptors and Signal Transduction* **2002**, *22* (1–4), 267–281. https://doi.org/10.1081/RRS-120014601.

(4) Kostenis, E.; Degtyarev, M. Y.; Conklin, B. R.; Wess, J. The N-Terminal Extension of Gαq Is Critical for Constraining the Selectivity of Receptor Coupling. *Journal of Biological Chemistry* **1997**, *272* (31), 19107–19110. https://doi.org/10.1074/jbc.272.31.19107.

(5) Wolf, P.; Beck-Sickinger, A. G. The Ring Size of Monocyclic ET-1 Controls Selectivity and Signaling Efficiency at Both Endothelin Receptor Subtypes. *J Pept Sci* **2021**, *27* (7), e3325. https://doi.org/10.1002/psc.3325.

(6) Schwarz, D.; Junge, F.; Durst, F.; Frölich, N.; Schneider, B.; Reckel, S.; Sobhanifar, S.; Dötsch, V.; Bernhard, F. Preparative Scale Expression of Membrane Proteins in Escherichia Coli-Based Continuous Exchange Cell-Free Systems. *Nat Protoc* **2007**, *2* (11), 2945–2957. https://doi.org/10.1038/nprot.2007.426.

(7) Krug, U.; Gloge, A.; Schmidt, P.; Becker-Baldus, J.; Bernhard, F.; Kaiser, A.; Montag, C.; Gauglitz, M.; Vishnivetskiy, S. A.; Gurevich, V. V.; Beck-Sickinger, A. G.; Glaubitz, C.; Huster, D. The Conformational Equilibrium of the Neuropeptide Y2 Receptor in Bilayer Membranes. *Angewandte Chemie International Edition* **2020**, *59* (52), 23854–23861. https://doi.org/10.1002/anie.202006075.

(8) Schmidt, P.; Bender, B. J.; Kaiser, A.; Gulati, K.; Scheidt, H. A.; Hamm, H. E.; Meiler, J.; Beck-Sickinger, A. G.; Huster, D. Improved in Vitro Folding of the Y2 G Protein-Coupled Receptor into Bicelles. *Front Mol Biosci* **2017**, *4*, 100. https://doi.org/10.3389/fmolb.2017.00100.

(9) Kaiser, A.; Wanka, L.; Ziffert, I.; Beck-Sickinger, A. G. Biased Agonists at the Human Y1 Receptor Lead to Prolonged Membrane Residency and Extended Receptor G Protein Interaction. *Cell. Mol. Life Sci.* **2020**, *77* (22), 4675–4691. https://doi.org/10.1007/s00018-019-03432-7.

(10) Takegoshi, K.; Nakamura, S.; Terao, T. – Dipolar-Assisted Rotational Resonance in Magic-Angle Spinning NMR. *Chemical Physics Letters* **2001**, *344* (5–6), 631–637. https://doi.org/10.1016/S0009-2614(01)00791-6.

(11) Tang, T.; Tan, Q.; Han, S.; Diemar, A.; Löbner, K.; Wang, H.; Schüß, C.; Behr, V.; Mörl, K.; Wang, M.; Chu, X.; Yi, C.; Keller, M.; Kofoed, J.; Reedtz-Runge, S.; Kaiser, A.; Beck-Sickinger, A. G.; Zhao, Q.; Wu, B. Receptor-Specific Recognition of NPY Peptides Revealed by Structures of NPY Receptors. *Sci. Adv.* **2022**, *8* (18), eabm1232. https://doi.org/10.1126/sciadv.abm1232.

(12) Vogel, A.; Bosse, M.; Gauglitz, M.; Wistuba, S.; Schmidt, P.; Kaiser, A.; Gurevich, V. V.; Beck-Sickinger, A. G.; Hildebrand, P. W.; Huster, D. The Dynamics of the Neuropeptide Y Receptor Type 1 Investigated by Solid-State NMR and Molecular Dynamics Simulation. *Molecules* **2020**, *25* (23), 5489. https://doi.org/10.3390/molecules25235489.

(13) Yang, Z.; Han, S.; Keller, M.; Kaiser, A.; Bender, B. J.; Bosse, M.; Burkert, K.; Kögler, L. M.; Wifling, D.; Bernhardt, G.; Plank, N.; Littmann, T.; Schmidt, P.; Yi, C.; Li, B.; Ye, S.; Zhang, R.; Xu, B.; Larhammar, D.; Stevens, R. C.; Huster, D.; Meiler, J.; Zhao, Q.; Beck-Sickinger, A. G.; Buschauer, A.; Wu, B. Structural Basis of Ligand Binding Modes at the Neuropeptide Y Y1 Receptor. *Nature* **2018**, *556* (7702), 520–524. https://doi.org/10.1038/s41586-018-0046-x.

(14) Qi, X.; Liu, H.; Thompson, B.; McDonald, J.; Zhang, C.; Li, X. Cryo-EM Structure of Oxysterol-Bound Human Smoothened Coupled to a Heterotrimeric Gi. *Nature* **2019**, *571* (7764), 279–283. https://doi.org/10.1038/s41586-019-1286-0.

(15) Zhang, L.; Hermans, J. Hydrophilicity of Cavities in Proteins. *Proteins* **1996**, *24* (4), 433–438. https://doi.org/10.1002/(SICI)1097-0134(199604)24:4%253C433::AID-PROT3%253E3.0.CO;2-F.

(16) Scheer, A.; Fanelli, F.; Costa, T.; De Benedetti, P. G.; Cotecchia, S. The Activation Process of the alpha1B-Adrenergic Receptor: Potential Role of Protonation and Hydrophobicity of a Highly Conserved Aspartate. *Proc Natl Acad Sci U S A* **1997**, *94* (3), 808–813. https://doi.org/10.1073/pnas.94.3.808.
(17) Ballesteros, J.; Kitanovic, S.; Guarnieri, F.; Davies, P.; Fromme, B. J.; Konvicka, K.; Chi, L.; Millar, R. P.; Davidson, J. S.; Weinstein, H.; Sealfon, S. C. Functional Microdomains in G-Protein-Coupled Receptors. The Conserved Arginine-Cage Motif in the Gonadotropin-Releasing Hormone Receptor. *J Biol Chem* **1998**, *273* (17), 10445–10453. https://doi.org/10.1074/jbc.273.17.10445.
(18) Klauda, J. B.; Venable, R. M.; Freites, J. A.; O'Connor, J. W.; Tobias, D. J.; Mondragon-Ramirez, C.; Vorobyov, I.; MacKerell, A. D.; Pastor, R. W. Update of the CHARMM All-Atom Additive Force Field for Lipids: Validation on Six Lipid Types. *J. Phys. Chem. B* **2010**, *114* (23), 7830–7843. https://doi.org/10.1021/jp101759q.
(19) Hess, B.; Bekker, H.; Berendsen, H. J. C.; Fraaije, J. G. E. M. LINCS: A Linear Constraint Solver for Molecular Simulations. *Journal of Computational Chemistry* **1997**, *18* (12), 1463–1472. https://doi.org/10.1002/(SICI)1096-987X(199709)18:12%253C1463::AID-JCC4%253E3.0.CO;2-H.
(20) Abraham, M. J.; Murtola, T.; Schulz, R.; Páll, S.; Smith, J. C.; Hess, B.; Lindahl, E. GROMACS: High Performance Molecular Simulations through Multi-Level Parallelism from Laptops to Supercomputers. *SoftwareX*, 2015, *1–2*, 19–25. https://doi.org/10.1016/j.softx.2015.06.001.
(21) Berendsen, H. J. C.; van der Spoel, D.; van Drunen, R. GROMACS: A Message-Passing Parallel Molecular Dynamics Implementation. *Comput. Phys. Commun.*, 1995, *91*, 43–56. https://doi.org/10.1016/0010-4655(95)00042-E.
(22) Humphrey, W.; Dalke, A.; Schulten, K. VMD: Visual Molecular Dynamics. *Journal of Molecular Graphics* **1996**, *14* (1), 33–38. https://doi.org/10.1016/0263-7855(96)00018-5.
(23) Pettersen, E. F.; Goddard, T. D.; Huang, C. C.; Meng, E. C.; Couch, G. S.; Croll, T. I.; Morris, J. H.; Ferrin, T. E. UCSF ChimeraX: Structure Visualization for Researchers, Educators, and Developers. *Protein Sci.*, 2021, *30*, 70–82. https://doi.org/10.1002/pro.3943.
(24) Monoz, M.; Sautel, M.; Martinez, R.; Sheikh, S. P.; Walker, P. Characterization of the Human Y1 Neuropeptide Y Receptor Expressed in Insect Cells. *Molecular and Cellular Endocrinology* **1995**, *107* (1), 77–86. https://doi.org/10.1016/0303-7207(94)03427-U.
